# An FtsH Protease Is Recruited to the Mitochondrion of *Plasmodium falciparum*

**DOI:** 10.1371/journal.pone.0074408

**Published:** 2013-09-13

**Authors:** Aiman Tanveer, Stacey M. Allen, Katherine E. Jackson, Manish Charan, Stuart A. Ralph, Saman Habib

**Affiliations:** 1 Division of Molecular and Structural Biology, CSIR-Central Drug Research Institute, Lucknow, India; 2 Department of Biochemistry and Molecular Biology, Bio21 Molecular Science and Biotechnology Institute, the University of Melbourne, Victoria, Australia; Institut national de la santé et de la recherche médicale - Institut Cochin, France

## Abstract

The two organelles, apicoplast and mitochondrion, of the malaria parasite *Plasmodium falciparum* have unique morphology in liver and blood stages; they undergo complex branching and looping prior to division and segregation into daughter merozoites. Little is known about the molecular processes and proteins involved in organelle biogenesis in the parasite. We report the identification of an AAA+/FtsH protease homolog (*Pf*FtsH1) that exhibits ATP- and Zn^2+^-dependent protease activity. *Pf*FtsH1 undergoes processing, forms oligomeric assemblies, and is associated with the membrane fraction of the parasite cell. Generation of a transfectant parasite line with hemagglutinin-tagged *Pf*FtsH1, and immunofluorescence assay with anti-*Pf*FtsH1 Ab demonstrated that the protein localises to *P. falciparum* mitochondria. Phylogenetic analysis and the single transmembrane region identifiable in *Pf*FtsH1 suggest that it is an i-AAA like inner mitochondrial membrane protein. Expression of *Pf*FtsH1 in *Escherichia coli* converted a fraction of bacterial cells into division-defective filamentous forms implying a sequestering effect of the *Plasmodium* factor on the bacterial homolog, indicative of functional conservation with *Ec*FtsH. These results identify a membrane-associated mitochondrial AAA+/FtsH protease as a candidate regulatory protein for organelle biogenesis in *P. falciparum*.

## Introduction

*Plasmodium* spp., the causal agents of malaria, contain two endosymbiotic organelles- a mitochondrion, and a relic plastid called the apicoplast. Each *Plasmodium* cell has a single apicoplast and mitochondrion with both organelles carrying their own reduced genomes [1,2,3,4,5,6]. The apicoplast is surrounded by four membranes, a result of its secondary endosymbiotic origin [7] and exists in close proximity with the parasite mitochondrion. The apicoplast is indispensable for parasite survival [8,9] and is the site of several biochemical pathways including type II fatty acid biosynthesis (FASII) [10,11], non-mevalonate synthesis of isoprenoid precursors [12,13], the SUF pathway of [Fe-S] cluster synthesis, and synthesis of haem [14,15,16]. The mitochondrion harbours the electron transport chain [17,18,19,20] and other pathways like the Isc like system for [Fe-S] cluster assembly [21], the initiation of haem biosynthesis [22] and of pyrimidine biosynthesis [23].

During asexual division of a parasite cell, the apicoplast and mitochondria are divided and segregated into daughter merozoites so that each daughter cell inherits a single copy of the organelle. The two organelles remain in close association with each other throughout erythrocytic development with visible contact points [24,25] although such an association does not seem to be necessary at least in the early exoerythrocytic liver stages [26]. Elegant live cell imaging has shown that apicoplasts are usually rounded in shape in the early erythrocytic stages, elongate during early schizogony, and branch at the late stages prior to segregation into daughter merozoites [11,24]. The mitochondria are elongated or branched before erythrocytic schizogony with frequent contact points with the plasma membrane and attain a highly branched morphology in the late blood schizont stages [24]. These mitochondria often contain looped regions, where the organelle apparently fuses back upon itself. During the asexual liver and blood stages as well as during gametogenesis, the mitochondrion is a more extensive structure than the apicoplast [24,26,27]. Division of the apicoplast appears to take place prior to mitochondrial division in both liver and blood stages; a single apicoplast is observed to be associated with a branch of the mitochondrion and after mitochondrial division each apicoplast/mitochondrion pair localises near a nucleus of the schizont and segregates into a daughter merozoite.

The process of extensive organellar branching followed by segregation for organellar division in *Plasmodium* would require large-scale alterations in membrane protein composition and stability. We thus investigated possible candidates that may play a role in maintaining membrane integrity and mediating organellar segregation in *Plasmodium falciparum*. One such class is the *fts* (**f**ilamentation **t**emperature **s**ensitive) genes that are known to play an important role in bacterial cell division. *Fts* mutants cause a defect in septum formation and cytokinesis that generates multinucleate filaments [28,29]. FtsZ, which is a major player in chloroplast division, is not found in apicomplexan parasites including *Plasmodium* [30,31]; homologs of another member of the *Fts* family, *ftsH*, have been identified in the malaria parasite and in *Toxoplasma gondii* [32]. FtsH belongs to the AAA+ (**A**TPases **A**ssociated with various cellular **A**ctivities) family of metalloproteases [33]. It was discovered as a mutant responsible for the defective growth of *E. coli* [34,35]. FtsH proteins are found in prokaryotes as well as mitochondria and chloroplasts of eukaryotes. Proteins of this family participate in cellular activities like protein degradation, regulation of the cell cycle, protein translocation and organelle biogenesis [36,37]. Two types of mitochondrial AAA/FtsH proteases, m-AAA and i-AAA, exhibiting different topologies in the mitochondrial membrane have been identified in the inner membrane of yeast, human and plant mitochondria [38]. The i-AAA proteases span the inner mitochondrial membrane and are exposed to the intermembrane space, while the m-AAA proteases have their active site exposed to the organellar matrix. Three plastid FtsH groups (P1, P2 and P3) have been described on the basis of sequence identity and hydropathy index [39,40].

Like all AAA+ family of proteins, FtsH has a conserved module of the ATPase domain encompassing Walker A, Walker B and the SRH (Second Region of Homology) motif [41]. The C-terminal region comprises the protease domain with a conserved Zn^2+^-binding metalloprotease active site ‘HEXGH’ [42] followed by a coiled-coil leucine zipper sequence [43]. FtsH is also of particular significance as it is the only AAA+ protease known to be essential for bacterial growth [44,45] and is the only membrane anchored *E. coli* protease of this family [46]. FtsH has a very weak protease activity and degrades proteins with very low thermostabilities [47]. The crystal structure of bacterial FtsH suggests that it exists as a homohexamer which is anchored to the membrane by the transmembrane domain. The hexamer forms a ring-like structure with a central pore [48,49]. In general, AAA+ proteases degrade their substrate protein by unfolding and translocating it through the central pore of the hexameric ring where the protease-active site exists at the pore wall. Typically, AAA+ proteases degrade substrates in a processive manner with no intermediates being formed [50].

We report characterization of a *P. falciparum* FtsH homolog that targets to the mitochondrion. *Pf*FtsH exists as a membrane-associated oligomeric complex in the parasite and is a Zn^2+^-dependent protease. A cytokinesis defect observed upon expression of recombinant *Pf*FtsH in *E. coli* suggests a role for the protein in mitochondrial biogenesis and division in *Plasmodium* .

## Materials and Methods

### Parasite culture

The *Plasmodium falciparum* strains (3D7 and D10_leader_ ACP-GFP) were cultured in human red blood cells (RBCs). RPMI 1640 (Sigma) media supplemented with 0.5% Albumax (Invitrogen) was used for culture maintenance. Parasite genomic DNA used as template for PCR was isolated by phenol-chloroform extraction.

### Ethics statement

The use of human RBCs from healthy volunteers for *P. falciparum* culture was approved by the CSIR-CDRI Institutional Ethics Committee (Human Research) (# CDRI/IEC/CEM/21-07-2010). Written informed consent was obtained from voluntary donors for use of this sample in research.

### Cloning, Expression and Purification of Recombinant *Pf*FtsH

*P. falciparum* FtsH homolog PFL1925w was amplified from genomic DNA. According to the PlasmoDB annotation, the *P. falciparum* FtsH is a single exon gene encoding a ~101 kDa protein, and the RNA-seq data available for this locus does not indicate any additional splice products. The full-length FtsH gene was PCR amplified using the upstream (PFLf: 5’-CGCGGATCCAGCTCGAAGTATGACAACCAG-3’) and downstream (PFLr: 5’- CGGGTCGACGTCGCTCTTAAATATGTCAAATAAAAA-3’) primers carrying BamHI and SalI restriction enzyme sites (underlined), respectively. The gene was cloned in the pQE30, pGEX and pMAL-c2 expression vectors and *E. coli* expression host was co-transformed with the one of the constructs and the RIG plasmid. The RIG plasmid carries tRNA genes whose transcripts recognize rare codons for the amino acids (aa) R, I and G in the *P. falciparum* A+T-rich DNA expressed in *E. coli* thus increasing the yield of the recombinant protein [51]. Next, the region corresponding to the internal region of FtsH (*Pf*FtsH_int_, aa 1 to 678) was cloned in the pGEX vector. This covers the entire gene except the region encoding the C-terminal non-conserved region. PCR with *P. falciparum* genomic DNA as template was carried out using the upstream primer used for full length FtsH and the downstream primer 5’-CGCGTCGACTCTTTTTACAAATGAATCTGAATT-3’. The *E. coli* C41(DE3) expression host was co-transformed with the pGEX-*Pf*FtsH_int_ construct and the RIG plasmid. The *E. coli* C41(DE3) strain contains uncharacterized mutation(s), which allows expression of toxic recombinant proteins [52]. Primary culture was set up by inoculating a single colony from the transformed plate and secondary culture was set up at 30°C till the O.D. reached ~1. After induction with 0.5 mM IPTG, cultures were grown for 16 hours at 22°C. The protein was affinity purified using a glutathione agarose 4B column. The cultures were suspended in lysis buffer (50 mM Tris, 300 mM NaCl, 10% w/v glycerol, 0.5% NP40 and 10 mM β-mercaptoethanol). Sonicated samples were passed through the column and washed with the same buffer. Protein was eluted with lysis buffer containing 20 mM reduced glutathione.

The region incorporating both the ATPase and the protease domain (aa 115 to 612) was also PCR-amplified and cloned for expression in *E. coli*. For PCR amplification the forward primer 5’-CGCGGATCCATGGGTAATGAGAAAAATAAGAAGAGT-3’ and reverse primer 5’-CGCGTCGACTTTCTCATTTTTCTTTGTATCATTTTTTAA-3’ containing BamHI and SalI sites, respectively were used. The amplification product was cloned in pQE30 vector and co-transformed in *E. coli* TG1 expression host along with the RIG plasmid. The recombinant protein was affinity purified on Ni-NTA Superflow (Qiagen) followed by a second step of purification by cation exchange chromatography using HiTrap SP HP column (GE Healthcare) with linear NaCl concentrations.

### Generation of parasites carrying hemagglutinin-tagged FtsH

For C-terminal HA-tagging of *P. falciparum* FtsH for localization studies, the region corresponding to the last 702 bp was PCR amplified by forward primer 5’-GGAAGATCTGTTAAAAATGAAGAAAACTTGAATAAT-3’ and reverse primer 5’-GCACTGCAGCGTCGCTTTTAAATATGTCAAATAA-3’ containing BglII and PstI sites, respectively. The fragment was cloned into the pHA3 transfection vector incorporating three HA tags at the C-terminus. 3D7 parasites were transfected with the resulting vector and parasites with integration into the locus of interest were selected as described by Duraisingh et al. [53].

### Microscopy

For immunofluorescence assays, parasite-infected erythrocytes were fixed in PBS containing 4% (v/v) para-formaldehyde and 0.0075% (v/v) glutaraldehyde, washed in PBS, permeabilised with 0.1% (v/v) Triton X-100 in PBS for 10 min at room temperature and washed with PBS as described by Tonkin et al. [54]. The HA-tagged FtsH was labeled using rat anti-HA monoclonal antibody 3F10 (1/100, Roche) and mouse anti-HA monoclonal antibody 12CA5 (1/400, Roche), and detected using Alexa Fluor 488- or 546-conjugated Goat anti-Rabbit/Rat/Mouse IgG (1:200, Molecular probes) for 1 hour at room temperature. Co-localisation experiments were performed with 20 nM Mitotracker Red CMXRos (Invitrogen), to detect the mitochondria, or with rabbit anti-Acyl Carrier Protein (a kind gift from Prof. G.I. McFadden), a marker of the apicoplast. Microscopy was performed using a Zeiss Axioplan2 using an Axio- CamMR camera, and with a DeltaVision Elite imaging system (API or an inverted Leica TCS-SP2 confocal microscope using a 63X oil immersion objective.

Immunofluorescence assays using the anti-FtsH antibody were carried out using *P. falciparum* 3D7 cells that were stained with Mitotracker Red CMXRos (Invitrogen), and fixed and permeabilised as above. Cells were probed with anti-FtsH1 antibody (1:20) followed by detection with anti-rabbit Alexa Fluor 514-tagged Ab. Samples were scanned in a Zeiss LSM510 confocal microscope using a 63X oil immersion objective.

### Antibody production and immunoblotting

#### Ethics statement

Institutional Animal Ethics Committee of the Central Drug Research Institute, India gave approval for the animal immunisation (#IAEC/2007/126/Renew02). Maintenance and care of animals was in accordance with Government of India guidelines.

Antibody was raised against recombinant *Pf*FtsH ATPase and protease domain (~57 kDa) of the protein in both rabbit and mice. Affinity-purified protein was electrophoresed on preparatory SDS-PA gel followed by staining with Coomassie G-250. The expected band was excised and protein was electro-eluted. Protein emulsion was made in Freund’s complete adjuvant for subcutaneous immunisation in rabbit and mice. Two booster doses to rabbit and one to mice were given in incomplete adjuvant. The antibody was checked by probing lysate of *E. coli* cells expressing the protein as well as parasite lysate with pre-immune and the immune sera.

For preparing parasite lysate for western blotting, parasites were released from RBC by 0.05% saponin lysis followed by washing with 1X PBS. The pellet was suspended in 1X Laemmli buffer with protease inhibitor cocktail (GBiosciences, USA). The sample was separated on 10% SDS-PA gel, blocked with 5% dry-skimmed milk at 4°C overnight. The blot was probed with primary rabbit anti-FtsH Ab and secondary HRP-tagged goat anti-rabbit Ab (Calbiochem) and developed using a chemiluminescent detection system (Millipore).

### Pulse-chase Assay

Pulse-chase analysis of *Pf*FtsH in parasite culture was performed at the late trophozoite stage as described by van Dooren et al. [55]. Cells were washed with methionine and cysteine-free RPMI-HEPES medium (Sigma), and then labeled by incubation for 90 min in methionine and cysteine-free RPMI-HEPES medium containing Elegmix (^35^S-labeled methionine and cysteine) (BARC, India). The medium was supplemented with 5% Albumax and 5% heat-inactivated human serum. After labeling, cells were washed twice with incomplete media and resuspended in RPMI-HEPES medium containing 5% Albumax and 5% heat-inactivated human serum and distributed into four culture dishes. Cells were harvested by saponin lysis at 0, 1, 2.5 and 5 hours of chase. The parasite pellets were subjected to immunoprecipitation (IP) with anti-*Pf*FtsH antibody. The parasite pellet was washed in PBS, and lysed in 500 µl of IP lysis buffer (0.05 M Tris-HCl, pH 7.5, 1% Triton X-100, 0.6 M KCl) containing a protease inhibitor cocktail (GBiosciences) for 5 min at room temperature and then incubated for 30 min on ice. The lysed cells were centrifuged at 12,000 rpm for 5 min. For pre-clearing, the supernatant was incubated with 80 µl of 50% slurry of protein A-Sepharose CL-4B beads (GE Healthcare) swelled in IP wash buffer (0.05 M Tris-HCl, pH 7.5, 1% Triton X-100, 1 mM EDTA, 0.15 M NaCl, 0.25% w/v BSA) for 1 h at 4°C. 5 µl of purified rabbit anti-FtsH Ab was incubated with 60 µl of 50% Sepharose slurry for 1 h at 4°C and added to the pre-cleared lysate. In a parallel set, the same amount of rabbit pre-immune serum was taken as control. After further incubation for 2 h at 4°C, the beads were pelleted down and washed four times in IP wash buffer and two times in PBS. Proteins were eluted from the beads by boiling in non-reducing sample loading buffer for SDS-PAGE. After electrophoresis, the 10% SDS-PA gel was fixed and fluorographed using a fluorography reagent (Amplify, GE Healthcare). The gel was dried and exposed to X-ray film. Another set of samples was prepared similarly and loaded onto SDS-PA gel under reducing conditions (β-ME added to the loading dye).

### Chemical cross-linking

Protein oligomerisation was studied by cross-linking parasite proteins with dithio-bis (succinimidyl propionate) (DSP), a reduction sensitive, cell permeable cross-linker. Cross-linking was performed as described by Bullen et al. [56,57] with some modifications. *P. falciparum* culture was pelleted down at 2000 rpm for 15 min and washed with PBS. Cells were resuspended in PBS containing 2 mM DSP and incubated at room temperature for 30 min. Following incubation, the reaction was stopped with 25 mM Tris pH 7.5 for 15 min at room temperature. The parasites were released from RBC by saponin lysis and resuspended in 1X NRSB buffer (0.05 M Tris-Cl, 10% glycerol, 2 mM EDTA, 2% SDS and Bromophenol Blue) with increasing concentration of DTT and incubated at 80°C for 5 min. The samples were electrophoresed in SDS-PA gels followed by western blotting with rabbit anti-FtsH antibody.

### Blue Native PAGE

Blue Native PAGE was performed according to Schägger and Jagow [58]. Parasite pellets were resuspended in resuspension buffer (0.75 M ε aminocaprioic acid, 50 mM Bis-Tris pH7.0) containing either 1% or 0.25% Triton X-100 and mixed at 4°C for 30 min. The insoluble fraction was removed by centrifugation at 14000xg for 30 min at 4°C. Samples were prepared by addition of loading buffer (5% solution of Coomassie G in 0.5M ε aminocaprioic acid) to the supernatant, electrophoresed on a 6 to 12% gradient gel and transferred onto PVDF membrane. Electrophoresis buffers were prepared as described by Reisinger and Eichacker [59]. The blot was probed with anti-FtsH antibody.

### Solubility characteristics of *Pf*FtsH

Membrane fractionation was carried out as described by Spork et al. [60]. *P. falciparum* D10 ACP_leader_-GFP parasite pellets were lysed in lysis buffer (50 mM Tris–Cl, 2 mM EDTA, pH 7.4) for 30 minutes and centrifuged at 36,000xg for 30 min to remove soluble proteins. The pellet (Tris-insoluble fraction) was further incubated in carbonate buffer (0.1 M sodium carbonate buffer, 1 mM EDTA, pH 11) for 30 min and subjected to centrifugation at 36,000xg for 30 min to release extrinsic membrane proteins. The supernatant contained the carbonate-soluble fraction. For recovery of intrinsic membrane proteins, the Tris-insoluble pellet was subjected to treatment with 1% Triton X-100 in PBS for 30 min followed by centrifugation at 36,000xg for 30 min. Tris, carbonate and Triton X-100 soluble fractions were subjected to TCA precipitation. The TCA precipitated pellets and Tris, carbonate and Triton X-100 insoluble fractions were suspended in equal volumes of PBS. Equal sample volumes were loaded on an SDS-PA gel and followed by western blotting with anti-FtsH Ab. The blot was stripped and re-probed with anti-GFP Ab (Roche, 1:1000).

### ATP binding and Protease Assay

Protease activity was estimated by the degradation of substrate protein α-casein (Sigma). 0.5 µg/µl of *Pf*FtsH (ATPase and protease domain) was incubated with 0.25 µg/µl of casein in protease assay buffer (10 mM Tris-Cl, 10 mM MgCl_2_, 100 mM NaCl, 10 µM zinc acetate and 1 mM DTT) in the presence of 8 mM ATP at 37°C. To test the role of metal ion on protease activity, reactions were performed in the presence or absence of 1 mM EDTA. To test ATP hydrolysis-dependent protease activity, the assay was performed in the presence or absence of 8 mM ATP or its non-hydrolysable analog Adenylyl Imidodiphosphate (AMPPNP). The reaction was terminated at different time points by the addition of 1 X SDS sample loading buffer, samples were separated on SDS-PAGE followed by coomassie staining.

For investigating binding of ATP to *Pf*FtsH, fluorescence emission spectra of control and protein samples in the presence or absence of 1 mM ATP were generated by excitation at 280 nm (path-length 5 mm) in a fluorimeter (LS50B, PerkinElmer).

### Cytokinesis in *E. coli*

*E. coli* C41 cells were co-transformed with *Pf*FtsH_int_ (expressed as a GST-tagged protein cloned in the pGEX vector) or the pGEX vector, and the RIG plasmid. Cultures were grown at 37°C until the O.D. reached 0.6 and induced with 0.5 mM IPTG. Three hours after induction at 20°C, 1ml of culture was withdrawn and pelleted down. The pellet was washed twice in 1X TBS (50 mM Tris pH7.5, 150 mM NaCl). Cells were fixed in 0.5% gluteraldehyde for 10 min at room temperature, washed with 1X PBS, and stained with 1 µg/ml of 4’, 6-diamidino-2-phenylindole (DAPI) for 30 min at 37°C. After three washes with 1X PBS, stained bacteria were mounted on a glass slide with mounting media. Slides were viewed in a fluorescence microscope (Leica DMI6000B). The expression of *Pf*FtsH_int_ in transformants was checked by western blotting of the IPTG induced culture with anti-GST antibody.

### Phylogenetic analysis

FtsH-like sequences were assembled using sequence similarity searches and the OrthoMCL tool [61]. Multiple sequence alignments were performed using ClustalW [62] and adjusted by hand. Phylogenies were inferred using maximum likelihood analysis with the PhyML3.0 package [63].

## Results

### Homologs of FtsH in *Plasmodium* spp.

FtsH metalloproteases are ubiquitous in Bacteria and Eukarya and several FtsH homologs that localise exclusively in plastids or mitochondria have been identified in eukaryotes. In apicomplexan parasites *T. gondii* and *P. falciparum*, three homologs of FtsH each are encoded by the nuclear genome and at least one of the *T. gondii* proteins is targeted to the apicoplast [32]. The three *P. falciparum* FtsH homologs [PlasmoDB accession numbers PF3D7_1239700 (PFL1925w), PF3D7_1464900 (PF14_0616), PF3D7_1119600 (PF11_0203)] have conserved Walker A and B motifs, the SRH region and the HEXGH Zn^2+^binding motif (Figure S1 in File S1). The coiled-coil leucine zipper region is found in PF14_0616 but is poorly conserved in PF11_0203 and PFL1925w. Two hydrophobic stretches that may serve as transmembrane regions are predicted for PF11_0203 while PF14_0616 and PFL1925w have a single predicted transmembrane region each. The single transmembrane domain in PFL1925w is shown in Figure S2 (File S1). Targeting predictions for the apicoplast or mitochondria of *P. falciparum* were unclear for all three; no signal peptide was recognised at the N-terminus for any protein, although a positively charged N-terminal transit peptide-like sequence was identified in PF14_0616 and PF11_0203 by PlasmoAP [64].

**Figure 1:**
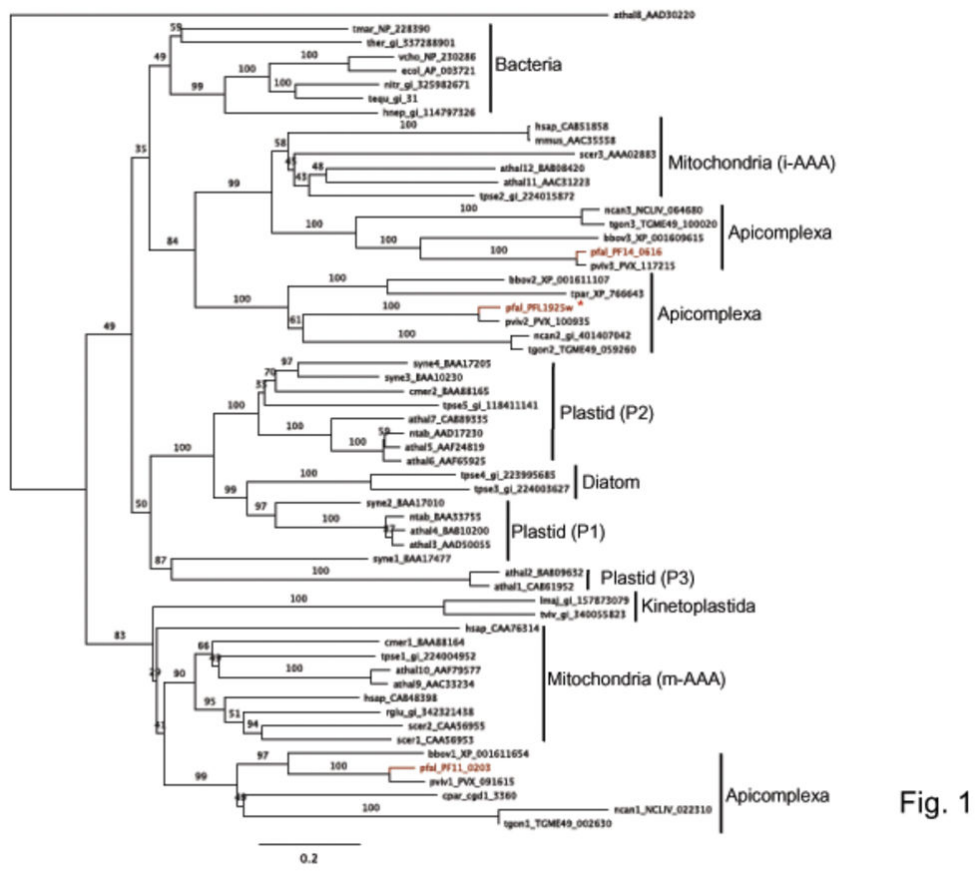
Phylogenetic tree of FtsH proteins from Bacteria, diatoms, yeast, alga, protozoa, plants and humans. Phylogenetic analysis using the maximum likelihood method indicates that the apicomplexan FtsH sequences form three distinct clades. Two of these (including the *Pf*FtsH analysed in this study, indicated by *) are closely allied with proteases known to face into the mitochondrial intermembrane space (i-AAA proteases), a third is allied with kinetoplastida proteases and proteases known to face into the mitochondrial matrix (m-AAA proteases). Athal, *Arabidopsis thaliana*; bbov, *Babesia* *bovis*; cmer, *Cyanidioschyzon* *merolae* ; cpar, *Cryptosporidium parvum*; ecol, *Escherichia coli*; hnep, *Hyphomonas* *neptunium* ; hsap, *Homo sapiens*; lmaj, *Leishmania major*; mmus, *Mus musculus*; ncan, *Neospora* *caninum* ; nitr, *Nitrosomonas* sp. AL212; ntab, *Nicotiana tabacum*; pfal, *Plasmodium falciparum*; pviv, *P. vivax*; rglu, *Rhodotorula* *glutinis* ; scer, *Saccharomyces cerevisiae*; syne, *Synechocystis* sp. PCC 6803; tequ, *Taylorellaequigenitalis* ; tgon, *Toxoplasma gondii*; ther, *Thermodesulfobacterium* sp. OPB45; tmar, *Thermotoga maritima*; tpse, *Thalassiosira pseudonana*; tviv, *Trypanosoma* *vivax* ; vcho, *Vibrio cholerae*. Sequences used in the alignment are available in File S2.

To understand the cellular distribution of FtsH proteases in *P. falciparum*, we carried out phylogenetic analysis of FtsH homologs from bacteria, diatoms, yeast, alga, protozoa, plants and mammals. Apicomplexan FtsHs formed three distinct clusters. The first, including the two-TMD PF11_0203 encompassed FtsHs from kinetoplastids as well as other mitochondrial matrix (m-AAA) FtsH homologs with two transmembrane domains (Figure 1). The other two *P. falciparum* FtsH proteins, PFL1925w and PF14_0616 possess only a single TMD, and cluster with inner mitochondrial membrane (i-AAA) FtsH homologues with a single TMD. No apicomplexan protein showed a clear alliance with FtsH proteins known to be involved in plastid division, although several diatom sequences appear good candidates for chromalveolate plastid division on the basis of their sequence similarity (Figure 1). Despite the apparent mitochondrial alliance of these apicomplexan proteins, PFL1925w grouped closely with a *Toxoplasma* protein, TGME49_059260, previously ascertained to be apicoplast-localised. Our interest in the biogenesis and segregation of the apicoplast in *P. falciparum* guided the selection of PFL1925w for further investigation and this protein is referred to as *Pf*FtsH1.

### Recombinant *Pf*FtsH1 and detection of the protein in *P. falciparum*

**Figure 2:**
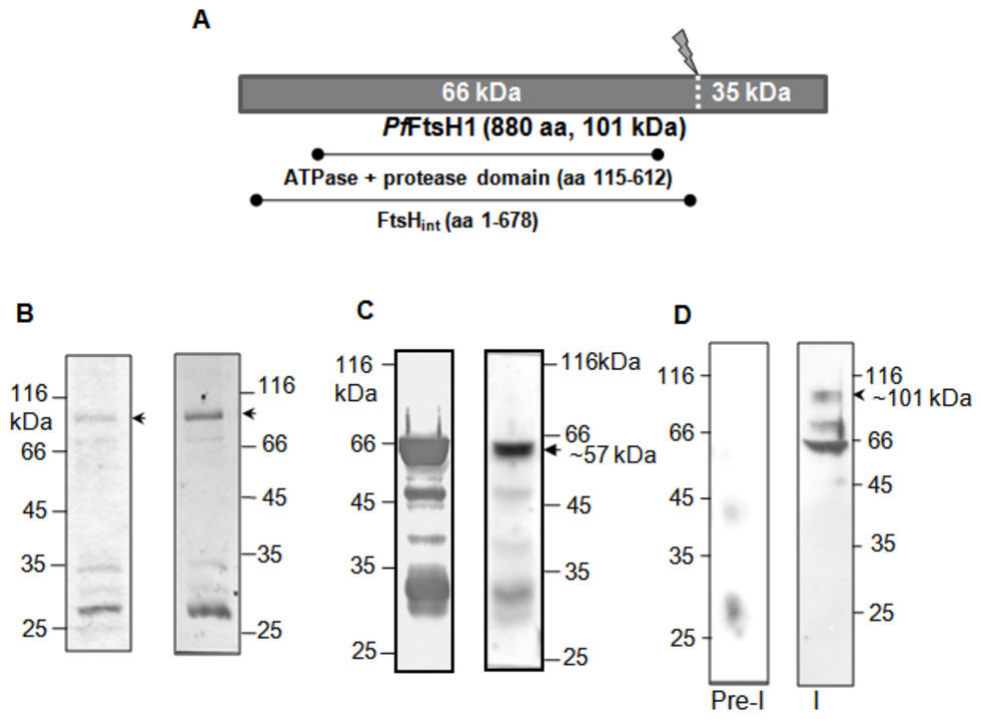
Recombinant expression of *Pf*FtsH1 in *E. coli* and its detection in the parasite lysate. (A) Line-drawing showing *Pf*FtsH1 stretches expressed in *E. coli* and the probable protein cleavage site. (B) Purified GST-*Pf*FtsH_int_ visualised in a coomassie-stained SDS-PA gel (left panel) and western blot analysis of purified protein using anti-GST Ab (right panel). (C) Purified His-*Pf*FtsH1 ATPase + protease domain on a coomassie-stained SDS-PA gel (left panel) and western blot of the protein with anti-His Ab (right panel). (D) *P. falciparum* lysate probed with anti-*Pf*FtsH1 Ab (I) detects a ~101 kDa band and a major ~66 kDa band. A minor band is also seen at ~72 kDa. No signal is detected with pre-immune serum (Pre-I).

For functional characterization of the protein, expression of full-length *Pf*FtsH1 was attempted in *E. coli* using different fusion tags (MBP, GST and His) at the N-terminus. Only the GST-tagged protein was expressed, albeit at very low levels and could not be purified. We then expressed the first 678 amino acids of *Pf*FtsH1 containing all the essential domains and excluding the nonconserved C-terminal region (Figure 2A). This GST-tagged protein (*Pf*FtsH_int_) of 104 kDa was expressed in *E. coli* to low levels (Figure 2B). This was used for complementation assays and for investigating effects of *Pf*FtsH1 expression in *E. coli*. The conserved ATPase and protease domain (aa115 to 612) of *Pf*FtsH1 was also expressed. This 57 kDa, N-terminal 6X-His tagged protein was purified in two steps. Western blotting showed the presence of the main 57 kDa band together with major degradation products of ~47 kDa and 30 kDa (Figure 2C). The 57 kDa band was electro-eluted and used to generate antibodies in rabbit. Rabbit immune serum was checked by probing bacterial lysate (data not shown) as well as the *P. falciparum* lysate (Figure 2D) with both pre-immune and immune sera. A band of ~101kDa which corresponds to the predicted size of full length *Pf*FtsH1 was detected in the parasite (Figure 2D). However, the most intense band was of ~66 kDa and might represent a cleavage/degradation product of the full length 101 kDa band (Figure 2A). The products of this cleavage and their detection is discussed in greater detail in subsequent sections of the manuscript. An additional band of ~72 kDa was occasionally detected in some western blots of parasite lysate.

### *Pf*FtsH1 localises to the parasite mitochondria and associates with the organellar membrane

To determine whether *Pf*FtsH1 was targeted to parasite organelle(s), a thermolysin protection assay was performed. Parasites were differentially permeabilised with the detergents digitonin and Triton X-100 [65,66] followed by treatment with the protease thermolysin. The major 66 kDa *Pf*FtsH1 band was protected from thermolysin cleavage after digitonin permeabilization in the *P. falciparum* D10 ACP_Leader_-GFP line [11] (Figure S3 in File S1). Similar protection was also observed for the full-length protein (101 kDa) although the signals for the band were fainter (data not shown). Apicoplast lumen-targeted ACP-GFP was similarly protected after digitonin treatment. Both *Pf*FtsH1 and ACP-GFP were cleaved by thermolysin when cells were treated with 1% Triton X-100. The presence of EDTA, a chelator of the thermolysin cofactor, reduced protein cleavage in the Triton X-100 treated samples. These results indicate that *Pf*FtsH1 localises to parasite organelles and is not accessed by thermolysin when cells are permeabilised with digitonin.

**Figure 3:**
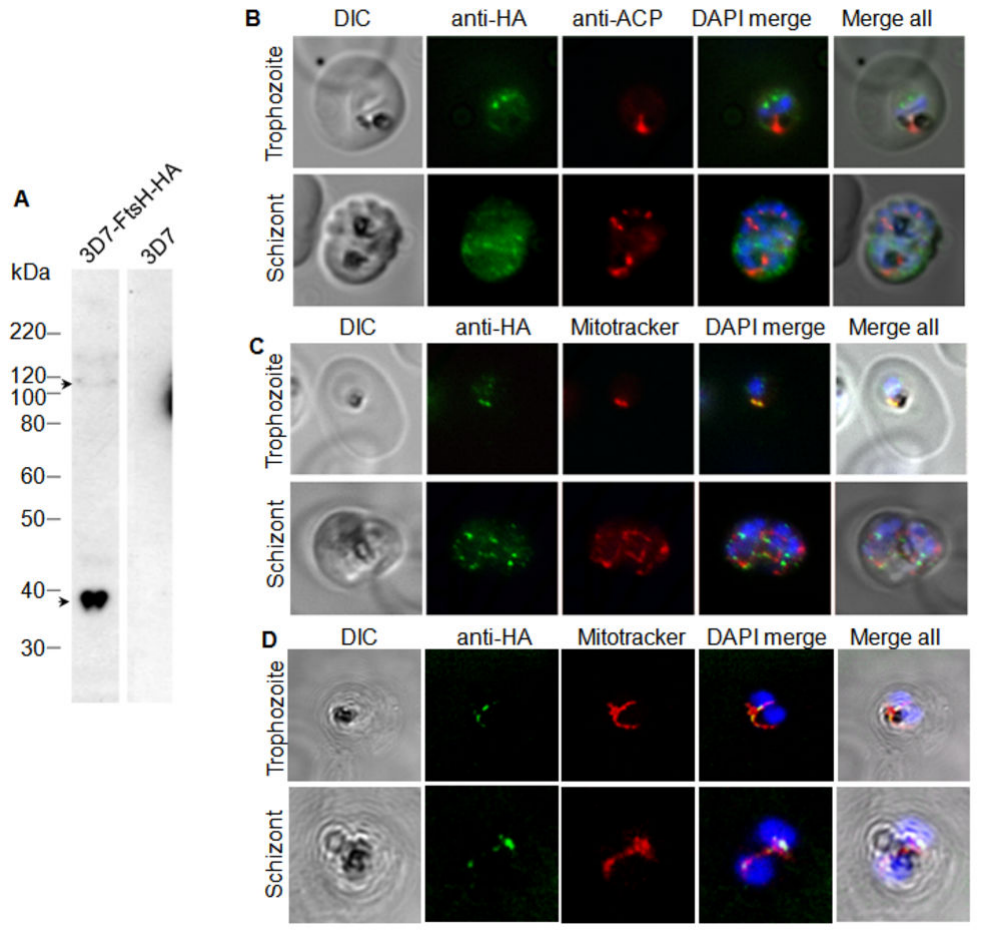
Localisation of *Pf*FtsH1 in a *P. falciparum* 3D7 transfectant line carrying C-terminal 3xHA-tagged *Pf*FtsH1. (A) Western with anti-HA mAb recognises an intact ~105 kDa (FtsH + HA tag) product and a ~38 kDa band likely to represent the cleaved ~35 kDa C-terminal region fused with HA. (B) Immunofluorescence localization of *Pf*FtsH1-HA using the anti-HA mAb and antibody against the apicoplast marker ACP. No overlap of *Pf*FtsH1-HA signal was observed with the apicoplast marker. (C) *Pf*FtsH1 co-localizes with the mitochondrial signal in trophozoites (upper panel) and appears as punctuate signals lining the organelle defined by the mitochondrial stain Mitotracker Red in schizonts (lower panel). (D) Confocal microscopy *Pf*FtsH1-HA expressing parasites showing co-localisation of *Pf*FtsH1 with the mitochondrion which is stained with Mitotracker Red.

**Figure 4:**
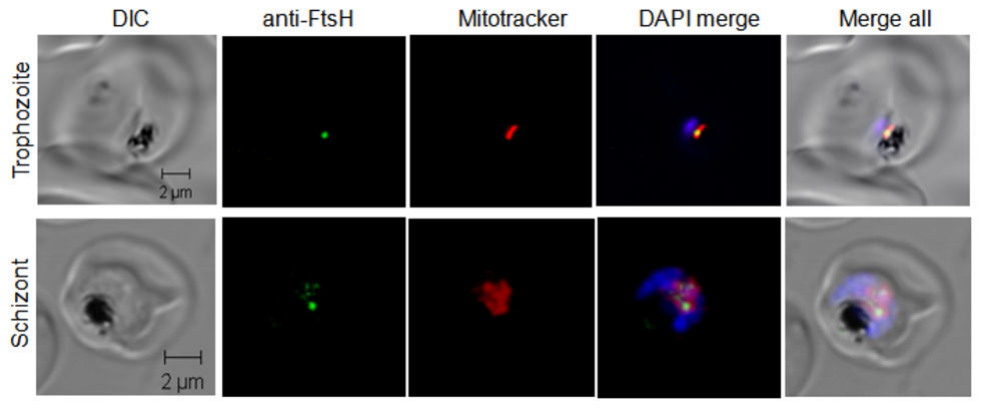
Localization of *Pf*FtsH1 to the mitochondrion is confirmed by immunofluorescence with anti-FtsH1 Ab. Confocal immunofluorescence microscopy of *P. falciparum* 3D7 infected erythrocytes using Mitotracker Red and anti-FtsH1 Ab shows localization of *Pf*FtsH1 in the parasite mitochondrion.

The partitioning of *Pf*FtsH1 to parasite apicoplast or mitochondria was further investigated by generating a C-terminal HA-tagged *Pf*FtsH1 line. The transfectants were selected and expression of the HA-tagged protein was checked by western blotting. A minor band of ~105 kDa (expected size of full-length FtsH + HA tag) and a prominent band of ~38 kDa (possibly a C-terminal cleavage product with the HA tag) were observed in western blots (Figure 3A). Immunofluorescence assays using anti-HA mAb and the antibodies against the apicoplast marker acyl carrier protein (ACP) did not show an overlap between the two signals indicating that *Pf*FtsH1 was not targeted to the apicoplast (Figure 3B). On the other hand, clear overlap was observed between the *Pf*FtsH1-HA signal and the mitochondria-specific dye Mitotracker Red both by wide-field and confocal fluorescence microscopy (Figure 3C and D, respectively). The major component of *Pf*FtsH1 recognised by the anti-HA mAb would be the 38 kDa C-terminal+HA segment along with the HA-tagged full-length protein. When the mitochondria elongated and branched in the schizont stages, punctuate signals of *Pf*FtsH1-HA were seen along the length of the organelle (Figure 3C, lower panel). Puncta of *Pf*FtsH1-HA were particularly prominent at mitochondrial branch points (movies S1 and S2). These results indicated that *Pf*FtsH1 is targeted to parasite mitochondria; we found no evidence for apicoplast targeting for this protein. The localisation of *Pf*FtsH1 to the mitochondrion was further confirmed by confocal microscopy using anti-FtsH1 Ab and Mitotracker Red (Figure 4).

**Figure 5:**
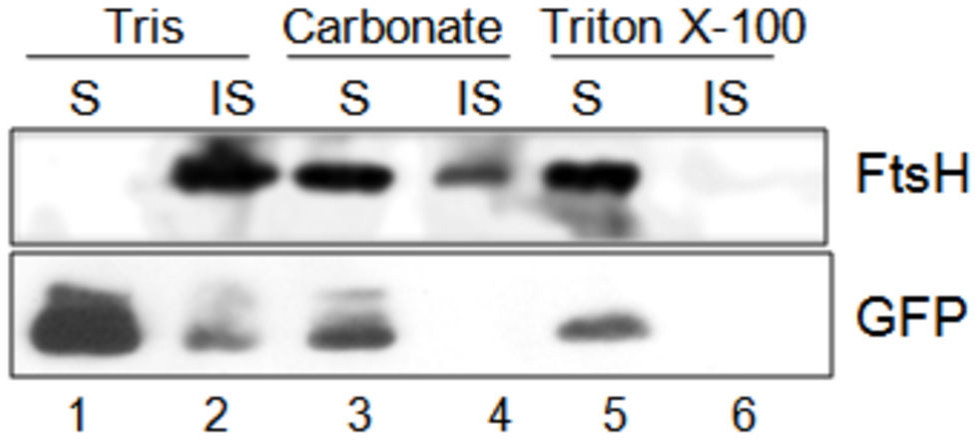
*Pf*FtsH1 is a membrane-associated protein. *P. falciparum* D10 ACP_leader_-GFP parasites were *s*equentially treated with Tris, sodium carbonate and Triton X-100 to investigate membrane association. Unlike apicoplast lumenal GFP, all *Pf*FtsH1 was Tris-insoluble and only partially solubilised by carbonate buffer, indicating membrane association. Almost all *Pf*FtsH1 was solubilised by Triton X-100. S, soluble fraction; IS, insoluble fraction.

FtsH in bacteria and plastids and mitochondria of eukaryotes is a membrane-bound metalloprotease. Association of *Pf*FtsH1 with membrane was investigated by sequential solubilisation of *P. falciparum* D10 ACP _leader-_GFP parasites with Tris followed by carbonate and Triton-X100 for removal extrinsic and intrinsic membrane proteins, respectively. Unlike apicoplast luminal GFP, *Pf*FtsH1 was completely insoluble in Tris buffer indicating membrane association (Figure 5). Although most *Pf*FtsH1 was solubilised by carbonate buffer, complete solubilisation was observed only upon treatment with Triton X-100. *Pf*FtsH1 is thus a membrane-associated protein. Interpreted with the localisation data above, *Pf*FtsH1 is identified as a mitochondrial membrane protein of *P. falciparum*. This is consistent with the phylogenetic grouping of this protein with other known mitochondrial membrane FtsHs.

### *Pf*FtsH1 is cleaved to a functional form in the malaria parasite

**Figure 6:**
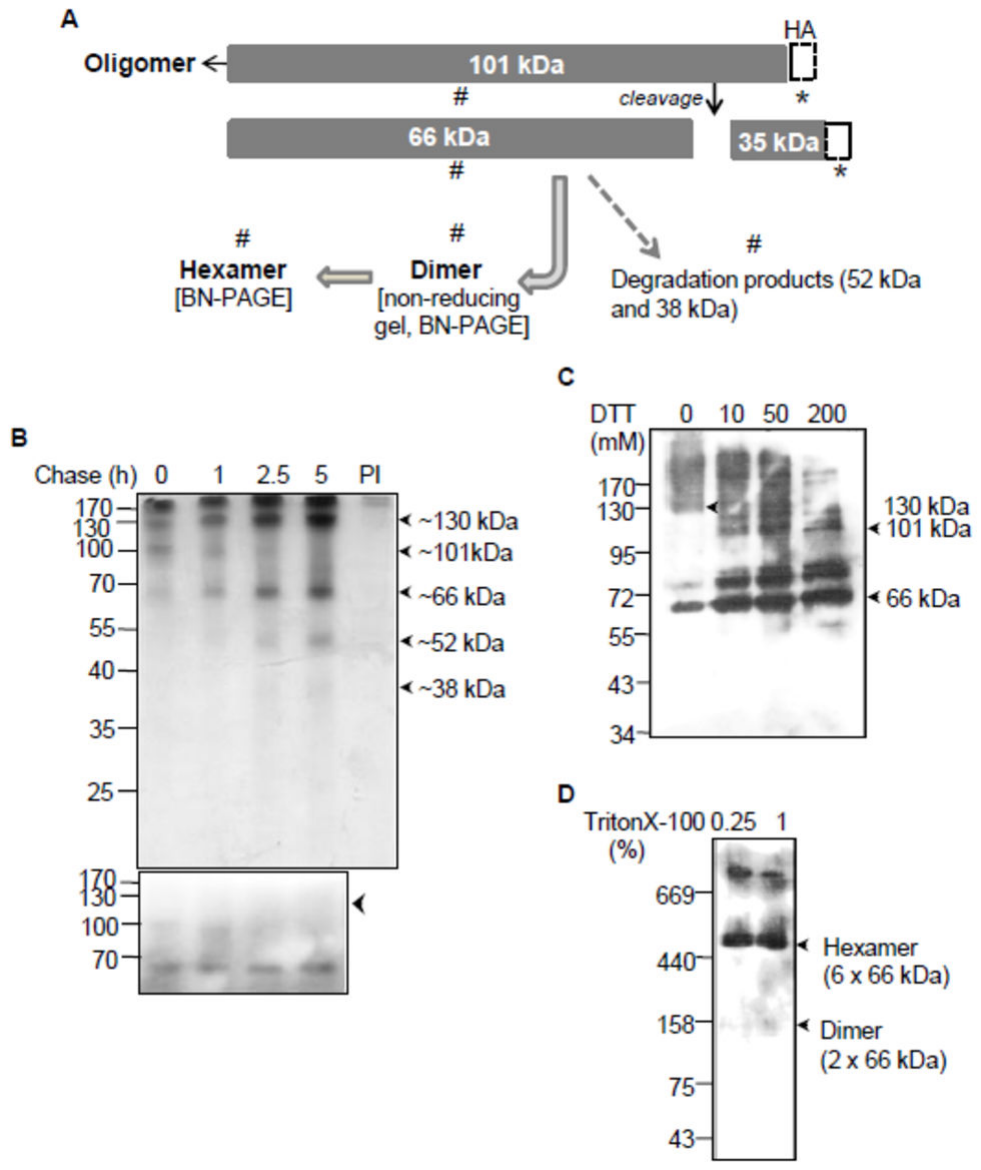
Processing and oligomeric assembly of *Pf*FtsH1 in the parasite. (A) Schematic of *Pf*FtsH1 processing inferred from results shown below and in Figure 2D and Figure 3A. * identifies *Pf*FtsH fragments detected by anti-HA mAb; # denotes fragments recognized by the anti-FtsH Ab generated against the ATPase+protease domain. (B) Pulse-chase of *P. falciparum*-infected erythrocytes followed by immunoprecipitation with anti-*Pf*FtsH antibody. Parasites at the early trophozoite stage were labeled with ^35^S methionine and cysteine for 90 min and harvested immediately after the pulse (0 h, lane 1) and after 1 h, 2.5 h and 5 h of chase (lanes 2-4). Lane 5 is immunoprecipitation at 0 h with pre-immune serum. A seven-day exposure of X-ray film was required to detect the signals above. (C) Parasite proteins were cross-linked in vivo by DSP followed by treatment with increasing concentrations of DTT to break the complex(s). *Pf*FtsH1 was detected by western blot analysis using anti-*Pf*FtsH1 Ab. (D) *Pf*FtsH1 exists in oligomeric complexes in the parasite as detected by BN-PAGE followed by western blotting with anti-*Pf*FtsH1 Ab.

*Pf*FtsH1 has a long C-terminal extension that lacks identity with known FtsH proteins, except *T. gondii* FtsH (TGME49_059260) that also has a long C-terminal extension that is removed by processing in the parasite [67]. The detection of a prominent 66 kDa band in western blots of *P. falciparum* lysates suggested the possibility of *Pf*FtsH1 being processed to a shorter functional form (Figure 2D). Processing of the protein was investigated by pulse-chase analysis. Pulse-chase of *Pf*FtsH1 carried out by metabolic labelling and immunoprecipitation with anti-*Pf*FtsH1 antibodies (Figure 6B, upper panel) revealed the presence of ~101 kDa band at the zero time point most of which was processed into a major 66 kDa band within 5 hours of chase. Increase in intensity of a 52 kDa and a faint 38kDa band was also observed suggesting that these were either degradation or specific cleavage products of the 66 kDa protein. No bands were detected by the control preimmune serum. In addition to the 101 kDa band, a 130kDa band was seen at the 0 h time point and its intensity increased until 2.5 h of chase. The accumulation of this protein during chase suggested the possibility of this being a dimer of the 66 kDa processed *Pf*FtsH1. To confirm this, pulse-chase analysis was performed under identical conditions except that immunoprecipitated samples were suspended in reducing gel-loading dye (Figure 6B, lower panel) as opposed to the non-reducing dye used in the gel shown in the upper panel. The 130 kDa band was not observed under reducing conditions suggesting that it represented a dimer of the 66 kDa processed *Pf*FtsH1 that dissociated in the reducing dye. Interestingly, the protein contains two cysteine residues in the conserved domains raising the possibility that either one or both residues may contribute to dimer formation through disulfide linkage(s).

The processing of the 101 kDa band into the 66 kDa product seen by pulse-chase analysis using the *Pf*FtsH1 antibody (Figure 6B), and detection of the 105 kDa band together with a 38 kDa band in western blot analysis of the *Pf*FtsH1-HA line (Figure 3A) indicates that a significant proportion of full-length *Pf*FtsH1 is processed into ~66 kDa and ~35 kDa products in the parasite. The 66 kDa band is detected only by the anti-FtsH1 Ab generated against recombinant *Pf*FtsH1 ATPase and protease domain in both western blots of *P. falciparum* lysate as well as immunoprecipitation and is not detected by the anti-HA mAb in western blots of the *Pf*FtsH1-HA line, indicating that it represents the N-terminal non-HA segment of the cleaved protein (Figure 6A). As expected, the anti-HA mAb detects the full-length protein and the 35 kDa C-terminal cleavage product fused to HA (total size of 38 kDa).

### FtsH1 exists as higher order oligomers in the parasite

To analyze oligomerization of *Pf*FtsH1 for understanding the architecture of the *Pf*FtsH1 complex in vivo, parasite proteins were chemically cross-linked by DSP to stabilize protein-protein interactions. This was followed by reduction of the cross-linked products by DTT. Addition of DTT resulted in the breakage of cross-linked components which were detected by western blotting with anti-*Pf*FtsH1 Ab (Figure 6C). In the absence of DTT (Figure 6C, lane 1), cross-linked complexes of ~130 kDa and >170 kDa together with the ~66 kDa band were detected. Increasing DTT concentration up to 200 mM released the ~101 kDa band and the major monomeric ~66kDa product, indicating that these *Pf*FtsH1 components in the cell exist as higher-order complexes. An additional band of ~75 kDa whose intensity increased with the addition of DTT was seen in all lanes and might represent a cross-linked product of 66 kDa *Pf*FtsH with an interacting partner whose linkage could not be broken by DTT.

The oligomeric status of *Pf*FtsH1 was further investigated by blue native PAGE. Parasites were treated with Triton X-100 to release the *Pf*FtsH1 complex from the membrane. Upon treatment with 0.25% and 1% Triton X-100, three complexes of 150 kDa, ~450 kDa and >700 kDa were detected by the anti-FtsH1 Ab (Figure 6D). The 150 kDa and 450 kDa bands are likely to represent dimeric and hexameric forms of the 66 kDa *Pf*FtsH1 but migrate at a slightly higher than expected size in the native PAGE. The identity of the uppermost complex is unclear; it might represent an oligomer formed by the full-length *Pf*FtsH1 or *Pf*FtsH1 complexed with its interacting partners.

### Recombinant FtsH1 exhibits zinc- and ATP-dependent protease activity

**Figure 7:**
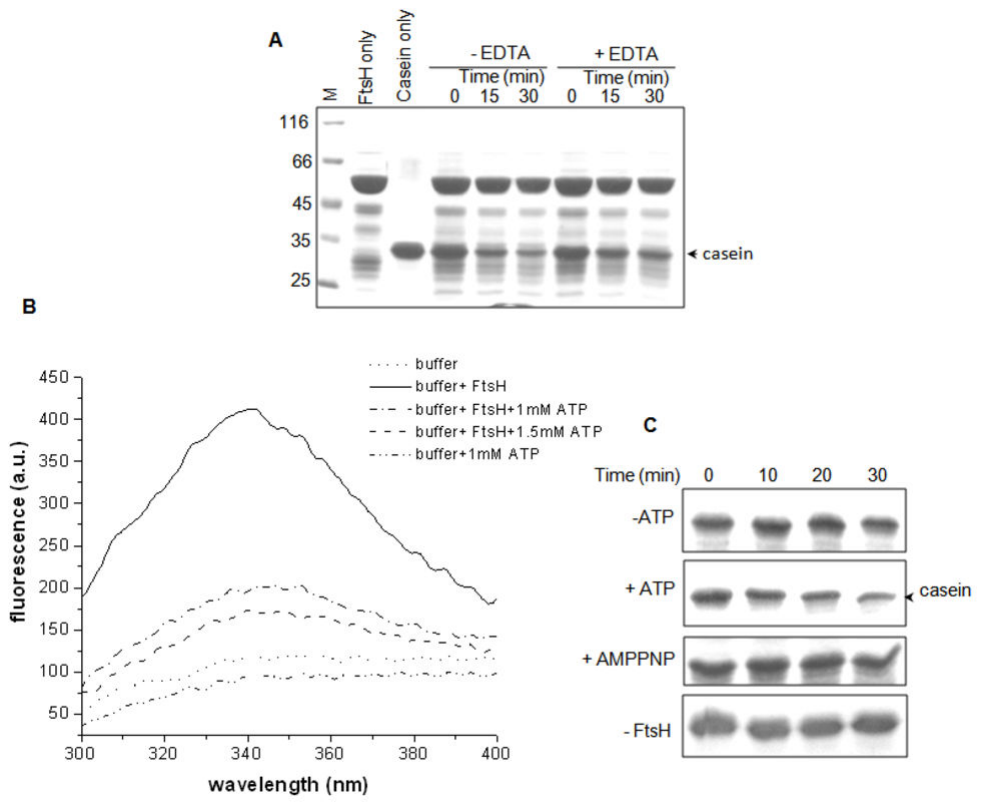
ATP- and Zn^2+^-dependent protease activity of *Pf*FtsH1. (A) *Pf*FtsH1 cleaves α-casein in a time-dependent manner in the presence of zinc and ATP. Addition of EDTA inhibits protease activity of *Pf*FtsH1 indicating the requirement of Zn^2+^ for *Pf*FtsH1-catalysed proteolysis. (B) *Pf*FtsH1 binds ATP as indicated by quenching of intrinsic fluorescence from the single tryptophan residue of the recombinant protein upon incubation with ATP. (C) ATP hydrolysis, and not just binding of the nucleotide to *Pf*FtsH1, is required for proteolytic activity. Proteolysis of α-casein was measured in the presence of ATP or its non-hydrolysable analog AMPPNP, and control sets lacking nucleotide or the enzyme. Proteolysis was observed only in the presence of ATP.

The protease activity of *Pf*FtsH1 was investigated by a proteolytic assay using the 57 kDa recombinant ATPase and protease domain of the protein. FtsH1 is a weak protease and degrades loosely folded proteins such as α-casein, which was taken as the substrate in the assay [47]. Time-dependent proteolysis of casein was observed in the presence of *Pf*FtsH1 (Figure 7A). Reduction of proteolysis was observed upon addition of EDTA indicating that a divalent cation (such as Zn^2+^) was required for *Pf*FtsH activity (Figure 7A). We were unable to detect ATPase activity of the purified 57 kDa *Pf*FtsH1 by the malachite green and EnzChek assays (Invitrogen) suggesting that it is a very weak ATPase. Binding of ATP to the protein was thus investigated by monitoring fluorescence changes upon ATP binding by measurement of intrinsic tryptophan fluorescence of the protein (the recombinantly expressed ATPase and protease domain of *Pf*FtsH1 has a single Trp residue) (Figure 7B). Quenching of intrinsic fluorescence upon incubation with ATP indicated alteration in protein conformation and/or masking of the Trp residue in the presence of the nucleotide, thus showing that *Pf*FtsH1 binds ATP.

Although catalysis of substrate protein degradation by FtsH requires Zn^2+^ and ATP hydrolysis [50], there are some conflicting views about the obligatory coupling of peptide hydrolysis with the hydrolysis of ATP; it has been suggested that nucleotide binding might suffice for cleavage of short peptides by FtsH [68,69]. A consensus view supports the notion that since the proteolytic reaction itself is not energy driven, ATP hydrolysis may be required for correct presentation of the protein substrate to the active site [70]. We investigated the requirement of ATP hydrolysis for substrate degradation by *Pf*FtsH1 by using AMPPNP, a non-hydrolyzable analog of ATP, in the proteolysis assay (Figure 7C). While casein degradation was observed in the presence of ATP, *Pf*FtsH1 was unable to degrade the substrate in the presence of AMPPNP. No degradation was observed in control sets lacking ATP or *Pf*FtsH1. Although *Pf*FtsH1 seems to be a weak ATPase, nucleotide hydrolysis and not just binding is required for *Pf*FtsH1-catalysed proteolysis.

### *Pf*FtsH1 causes defective cytokinesis in *E. coli*

Considering the conservation between important domains in the *E. coli* FtsH and *Pf*FtsH1 we attempted in vivo complementation of *Ec*FtsH function by GST-*Pf*FtsH_int_ using the *E. coli* AR423 strain (Δ*ftsH::kan/*pAR171) [71]. The pAR171 has the essential *EcftsH* gene under the *ftsH* promoter and a temperature sensitive *ori* that makes it defective for replication at 42°C. Although expressed in the *E. coli* AR423 host cell, GST-*Pf*FtsH_int_ did not complement *Ec*FtsH at the non-permissive temperature (Figure S4 in File S1). This may be attributed to differences in activity and/or substrate specificity of the *E. coli* and *Plasmodium* proteins. Another reason may be the reduced stability of full-length GST-*Pf*FtsH_int_ in *E. coli* AR423 at 42°C (Figure S4B in File S1).

**Figure 8:**
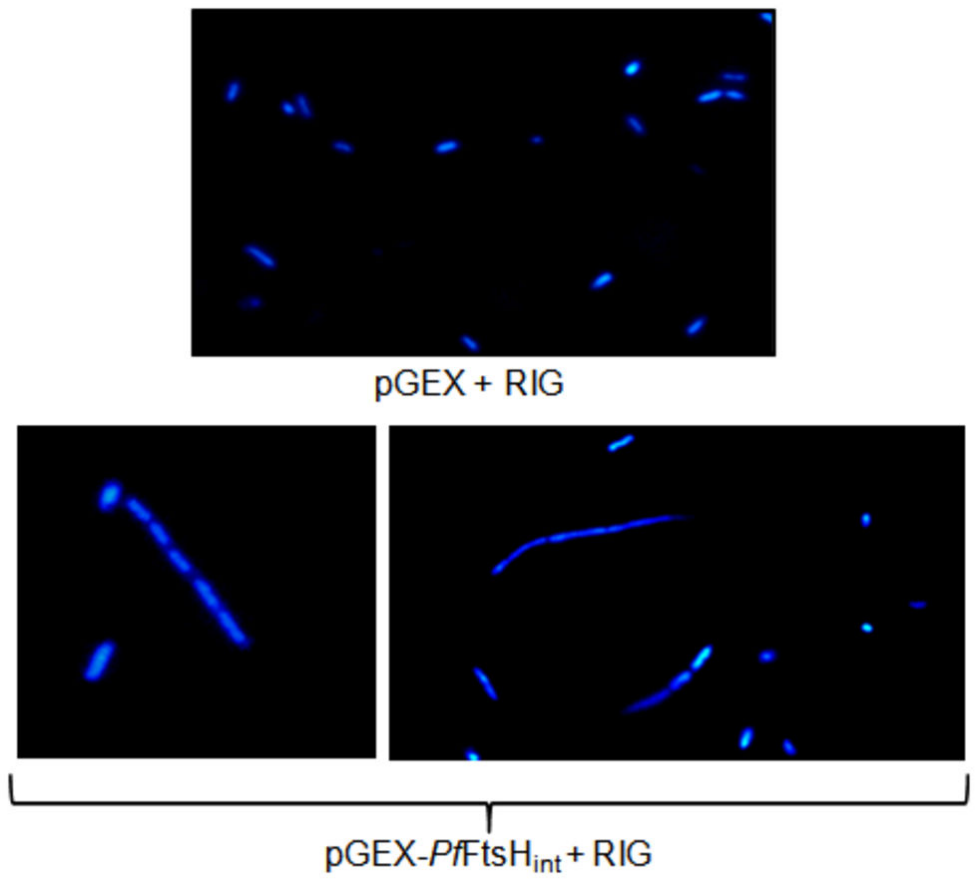
Defective cytokinesis observed in a fraction of *E. coli* cells expressing *Pf*FtsH_int_. *E. coli* cells transformed with pGEX + RIG or pGEX-*Pf*FtsH_int_ +RIG were grown for 3 h at 20°C after induction, fixed and stained with DAPI.

To further investigate the effect of *Pf*FtsH1 in *E. coli*, we evaluated changes in the growth and morphology of *E. coli* C41 cells expressing GST-*Pf*FtsH_int_. *E. coli* C41 cells transformed with pGEX-*Pf*FtsH_int_ and the RIG plasmid were compared with cells transformed with RIG and the pGEX vector alone. The former had a slower rate of growth compared to the latter. When DAPI-stained bacteria were examined in a fluorescence microscope, a fraction (~9.5% ± 3.6, number of fields=15, total cells counted=1017) expressing GST-*Pf*FtsH_int_ were elongated filaments with defective septum formation and cytokinesis while the control cells transformed with pGEX + RIG had normal morphology (Figure 8). This suggests an interaction between GST-*Pf*FtsH_int_ and *E. coli* FtsH such that the former sequesters or replaces the latter in higher order oligomeric complexes resulting in the inhibition of normal cytokinesis. Such antagonistic effect of GST-*Pf*FtsH_int_ is indicative of functional conservation and implies a role for *Pf*FtsH1 in organellar division.

## Discussion

Members of the FtsH family of Zn^2+^ metalloproteases perform diverse and essential functions in bacteria and organelles of eukrayotes. Homologs of FtsH are encoded by genomes of parasitic protozoa of the phylum Apicomplexa, but their functions in these parasites remain to be understood. Targeting and processing of an FtsH homolog of *T. gondii* has been reported [32]. In order to understand cellular functions of parasite FtsH proteins and their possible role in organellar function, we characterised the processing and function of an FtsH homolog of *P. falciparum*. Targeting of *Pf*FtsH1 to the parasite mitochondria, its association with the organellar membrane, ATP-dependent protease activity of the recombinant protein, and its effect on *E. coli* cytokinesis identified it as an AAA Zn^2+^-dependent protease involved in mitochondrial biogenesis.

Several FtsH orthologues localised exclusively in mitochondria or plastids have been identified in other organisms. Numbers range from the single FtsH of *E. coli* [72] to 12 active FtsHs encoded by *Arabidopsis thaliana* [73]. Out of these, eight are chloroplast-targeted [74], three are targeted to mitochondria, and one is dually targeted to both organelles [75]. FtsH/AAA protease complexes in yeast, human and plant mitochondria have two distinct topologies, i-AAA and m-AAA, and span the inner mitochondrial membrane. The i-AAA FtsH proteases have a single transmembrane region and expose their catalytic domains to the mitochondrial intermembrane space while the m-AAA proteases have two transmembrane regions with the catalytic domains directed towards the organellar matrix. Our phylogenetic analysis and transmembrane domain analysis suggests that the *Pf*FtsH characterised in this study (PFL1925w) and a second *P. falciparum* FtsH homolog, PF14_0616, are single TMD proteins grouping with the mitochondrial i-AAA type proteases. A *Toxoplasma* protein belonging to this group has previously been localised to the apicoplast, although this protein has a very different N-terminus to the *Plasmodium* homologues, and presumably contains different targeting information A third *P. falciparum* FtsH homolog, PF11_0203, is part of the apicomplexan FtsH cluster that forms a clade with mitochondrial m-AAA proteases. Like known m-AAA, PF11_0203 has two predicted transmembrane regions. Further localisation experiments are warranted to determine if any of the three *Plasmodium* FtsH like proteins are apicoplast resident.

FtsH/AAA+ proteases form hexameric oligomers and crystal structure of the cytosolic region of *Thermus thermophilus* FtsH shows that it exists as a trimer of dimers [48,76] that form a large hexameric ring. Western blotting, immunoprecipitation, BN-PAGE and in vivo cross-linking experiments show that *Pf*FtsH1 is processed to produce two forms – an N-terminal ~66 kDa form, and a C-terminal ~35 kDa form. The N-terminal version assembles into a dimer of ~130 kDa. The corresponding hexamer was also seen in the parasite by BN-PAGE. A band of ~101 kDa corresponding to the size of the full-length protein was also detected in *P. falciparum* 3D7 cells and *Pf*FtsH1-HA and a ~101 kDa band was released from crosslinked complexes after DTT treatment but it is unclear whether this is generated after cleavage of a mitochondrial targeting peptide. The related apicoplast-targeted *T. gondii* FtsH1 is processed by a protease at both the N- and C- termini, with N-terminal processing taking place in the ER [67] and *E. coli* FtsH has been demonstrated to undergo ATP-dependent C-terminal self-cleavage [77]. The mechanism of *Pf*FtsH1 cleavage remains to be determined but our results indicate that the ~35 kDa non-conserved region from the C-terminus is cleaved to generate the 66 kDa product.

A complex >700 kDa detected in BN-PAGE as well as higher complexes seen in DSP crosslinking indicate that the *Pf*FtsH1 hexamer forms a complex with interacting partners as also seen with FtsH proteins in other organisms. *E. coli* FtsH exists in the plasma membrane as a holoenzyme multiprotein complex of 1000 kDa comprising of hexamers made of FtsH monomers and a hexamer of HflKC pairs [78]. In *A. thaliana* mitochondrial m-AAA assemble with prohibitions to form a complex of ~2 MDa [79] and yeast mitochondrial m-AAA protease forms a super-complex with prohibitin [80]. Yeast i-AAA protease complexes with Mgr3p and Mgr1p which act as adapter proteins and regulate its protease activity [81]. We attempted to identify putative interacting partners of mitochondrial *Pf*FtsH1 by scanning the yeast-two-hybrid *P. falciparum* interactome data 2 proteins (PFI1075w and MAL13P1.102) listed as putative interaction partners of PFL1925w (*Pf*FtsH1) were cloned for recombinant expression in *E. coli*, of which only the expression of MAL13P1.102 was successful. MAL13P1.102 is annotated as a cytosolic protein of unknown function and our attempts to detect its interaction with *Pf*FtsH1 in vitro were unsuccessful (data not shown). Mitochondrial interacting partners of *Pf*FtsH1 remain to be identified.

AAA proteases of the mitochondrial inner membrane conduct protein surveillance by degrading non-native integral membrane proteins and membrane-associated proteins that include unassembled units of the respiratory chain complex [82,83,84]. m- and i-AAA proteases have overlapping substrate specificity. In addition to protein quality surveillance, they also have specific substrates whose proteolysis regulates central processes in the mitochondria. For instance, i-AAA protease determines the stability of two inter-membrane space proteins, Ups1 and Ups2, which regulate the biogenesis of the mitochondria-specific phospholipid cardiolipin as well as phosphatidylethanolamine [85]. The m-AAA protease also promotes maturation of specific proteins such as MrpL32, a component of the large subunit of the mitochondrial ribosome [86] thus promoting its assembly in the ribosome and activating mitochondrial translation. Interestingly, i-AAA protease in yeast can also promote import of heterologously expressed mammalian polynucleotide phosphorylase in a manner that is independent of its proteolytic activity thus suggesting an additional role for AAA proteases in translocation [87]. OPA-1, a human mitochondrial dynamin-like GTPase involved in mitochondrial fusion and maintenance of cristae morphology [88] undergoes complex cleavage in the inner membrane space and cristae junctions in the inner mitochondrial membrane; m-AAA and i-AAA proteases contribute to OPA-1 cleavage [89,90] thus participating in the regulation of mitochondrial shape. *Pf*FtsH1 is a membrane associated protein and exhibited punctuate distribution in the parasite mitochondria with concentration of signal in constricted regions and branch points in elongated mitochondria of the late trophozoite/early schizont stages (Figure 3 and movies S1 and S2). In dividing bacterial cells, FtsH accumulates at the mid-cell septum and plays a regulatory role in cell division [91]. Defective division of a fraction of bacterial cells upon expression of *Pf*FtsH1 showed an inhibitory effect of the parasite protein on host *E. coli* FtsH that is indicative of conservation of function between the two FtsHs. The defective cellular morphology is in agreement with the phenotype of *E. coli* cells expressing the red alga *Cyanidioschyzon* *merolae* *ftsH* gene where *C* *. merolae* FtsH disrupted cytokinesis and led to the formation of filamentous cells [92]. Thus *Pf*FtsH1 is likely to play a regulatory role in mitochondrial division. The specific target proteins of *Pf*FtsH1 remain to be identified.

We identify an AAA/FtsH protease that targets to the *P. falciparum* mitochondrion, is associated with the organellar membrane, and has similarity with mitochondrial inner membrane i-AAA proteases from other organisms. The ATP- and Zn^2+^-dependent protease is processed in the parasite and cellular oligomeric assemblies of the protein can be identified. Although future studies will concentrate on identification of *Pf*FtsH1 targets in the mitochondrion, our results provide early evidence for its role in division of the uniquely elongated and branched mitochondria in the erythrocytic stages of the malaria parasite.

## Supporting Information

File S1 (PPT)

File S2 (DOCX)

Movie S1 (AVI)

Movie S2 (AVI)
