# Supplementary material for: An FtsH Protease Is Recruited to the Mitochondrion of Plasmodium falciparum
Source: PLoS One. 2013 Sep 13;8(9):e74408. doi: 10.1371/journal.pone.0074408 (PMC3772908; doi:10.1371/journal.pone.0074408)
Supplement: File S1 — (PPT) [file pone.0074408.s001.ppt]

## Slide 1
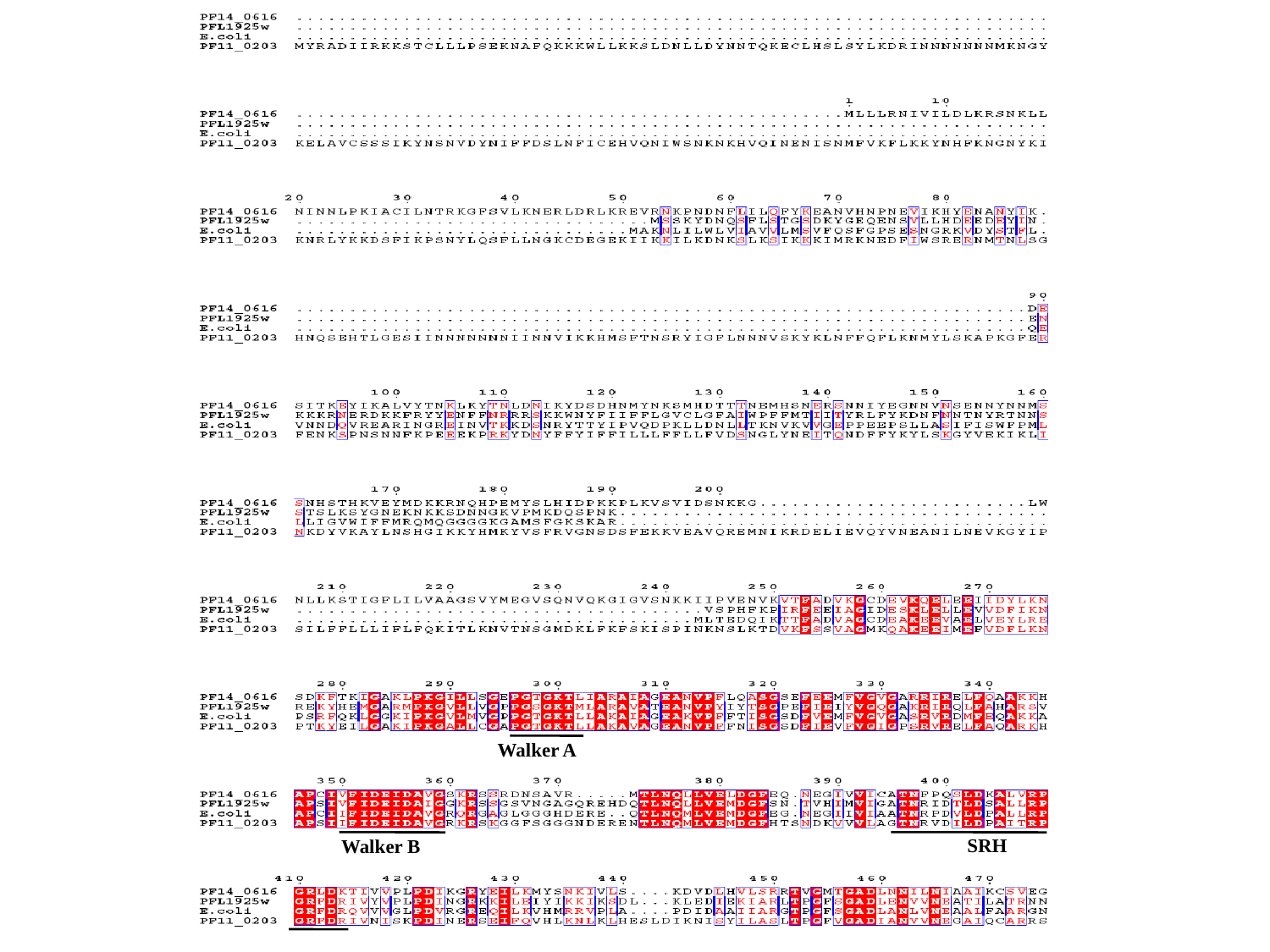

Walker A
SRH
Walker B

## Slide 2
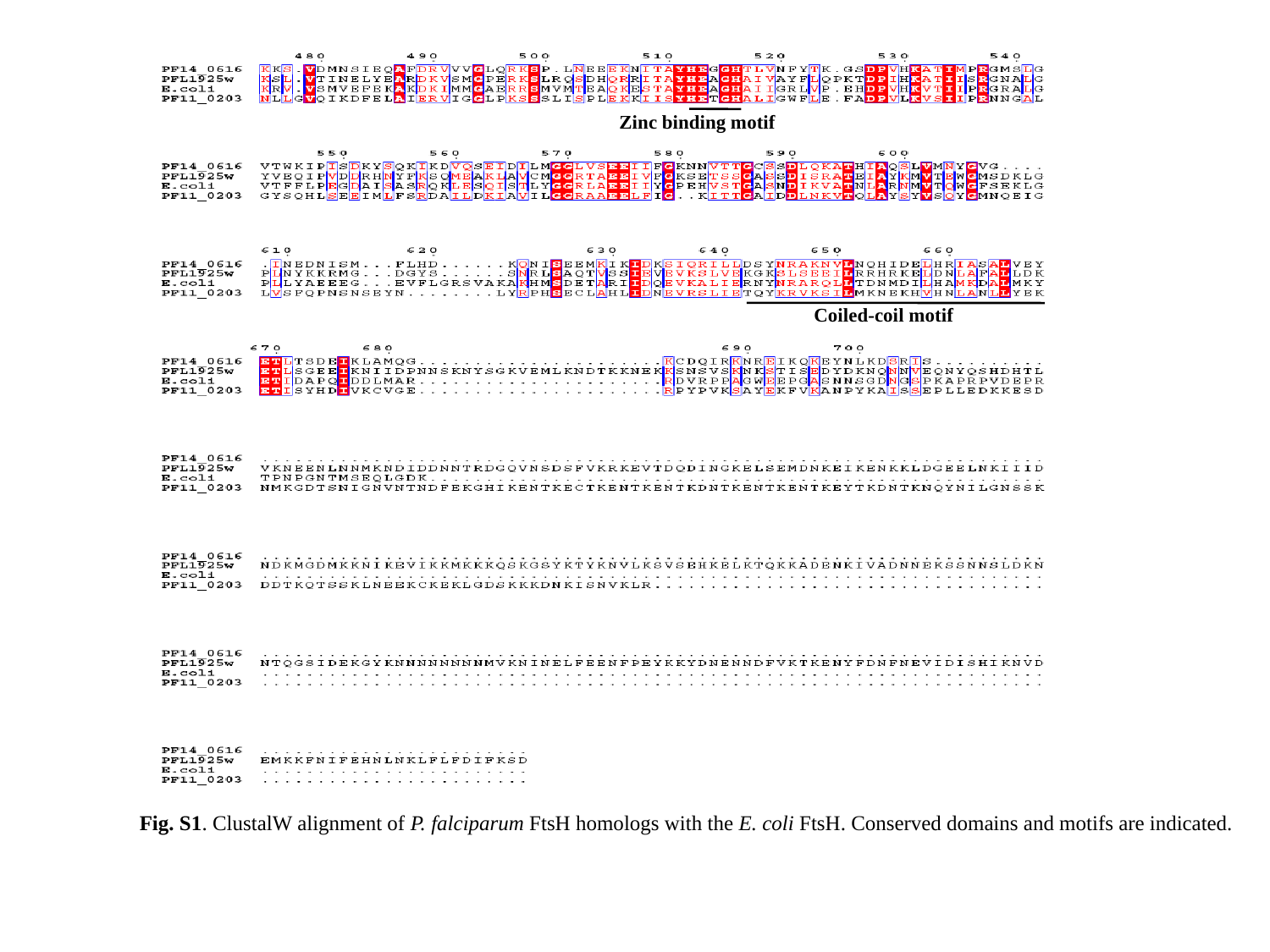

Zinc binding motif
Coiled-coil motif
Fig. S1. ClustalW alignment of P. falciparum FtsH homologs with the E. coli FtsH. Conserved domains and motifs are indicated.

## Slide 3
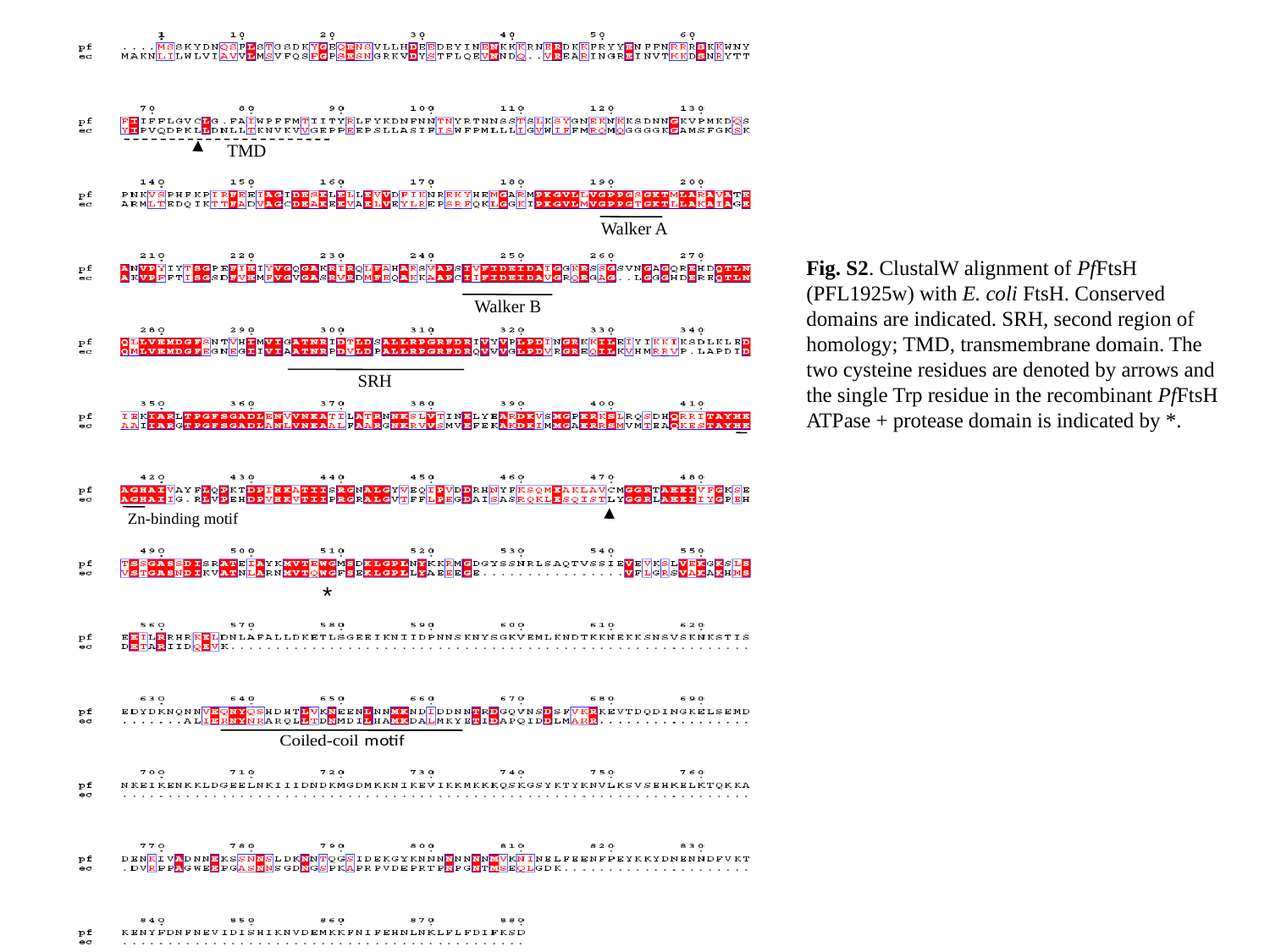

Walker A
Walker B
SRH
*
Zn-binding motif
TMD
Fig. S2. ClustalW alignment of PfFtsH (PFL1925w) with E. coli FtsH. Conserved domains are indicated. SRH, second region of homology; TMD, transmembrane domain. The two cysteine residues are denoted by arrows and the single Trp residue in the recombinant PfFtsH ATPase + protease domain is indicated by *.

## Slide 4
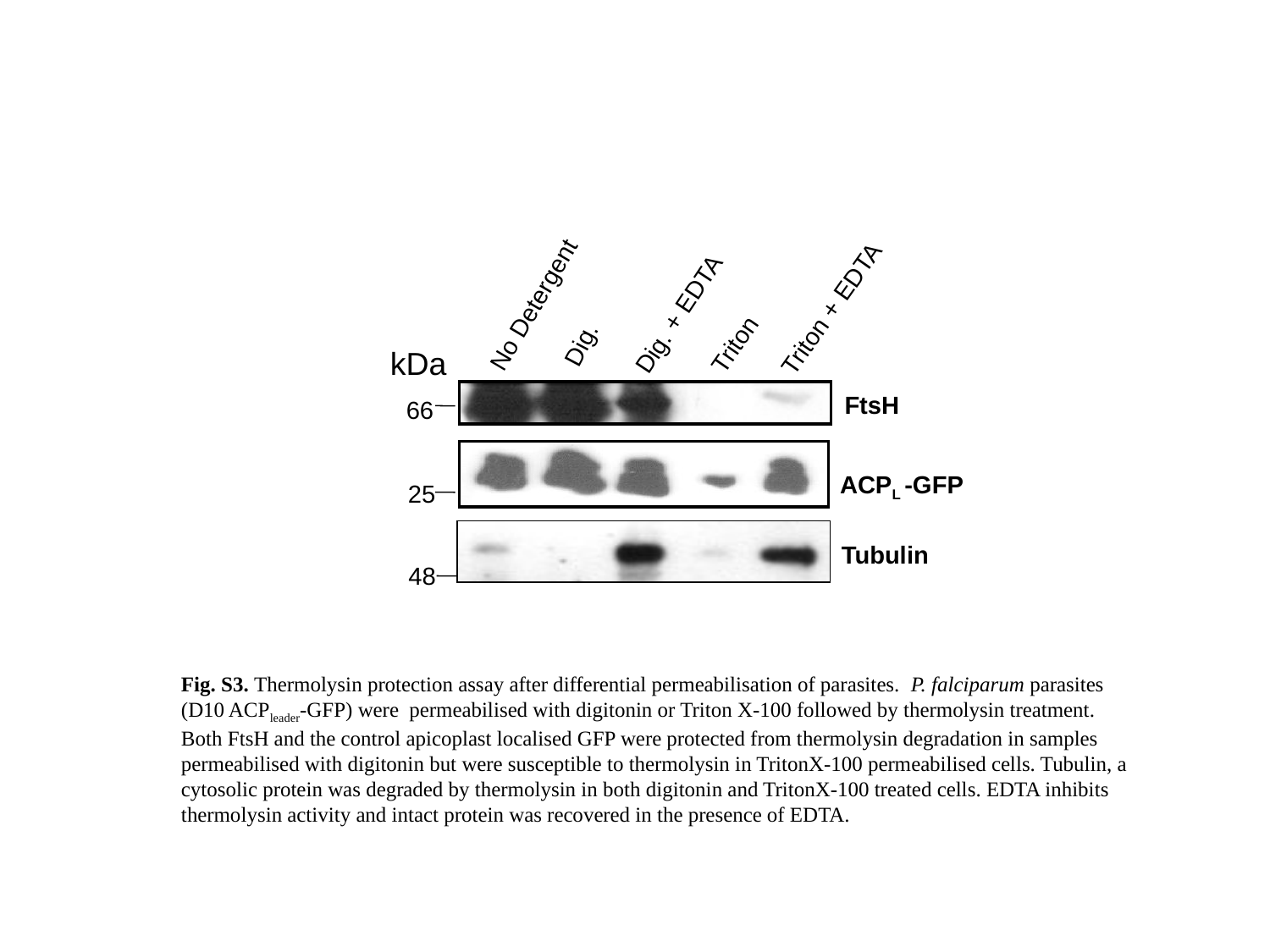

Triton
Dig.
Triton + EDTA
No Detergent
Dig. + EDTA
kDa
FtsH
66
ACPL -GFP
25
Tubulin
48
Fig. S3. Thermolysin protection assay after differential permeabilisation of parasites. P. falciparum parasites
(D10 ACPleader-GFP) were permeabilised with digitonin or Triton X-100 followed by thermolysin treatment.
Both FtsH and the control apicoplast localised GFP were protected from thermolysin degradation in samples permeabilised with digitonin but were susceptible to thermolysin in TritonX-100 permeabilised cells. Tubulin, a cytosolic protein was degraded by thermolysin in both digitonin and TritonX-100 treated cells. EDTA inhibits thermolysin activity and intact protein was recovered in the presence of EDTA.

## Slide 5
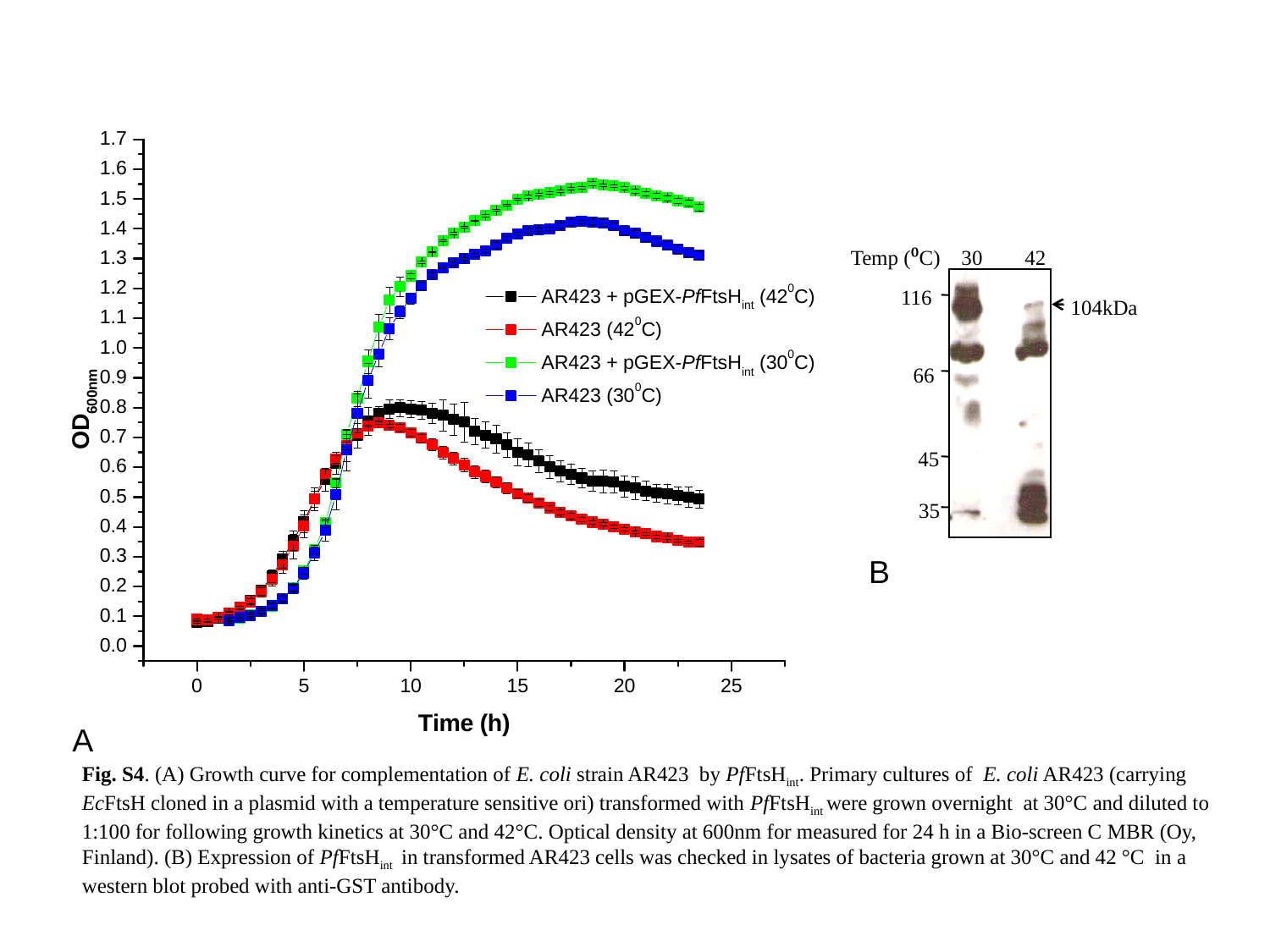

Temp (⁰C) 30 42
116
66
45
35
104kDa
B
A
Fig. S4. (A) Growth curve for complementation of E. coli strain AR423 by PfFtsHint. Primary cultures of E. coli AR423 (carrying EcFtsH cloned in a plasmid with a temperature sensitive ori) transformed with PfFtsHint were grown overnight at 30°C and diluted to 1:100 for following growth kinetics at 30°C and 42°C. Optical density at 600nm for measured for 24 h in a Bio-screen C MBR (Oy, Finland). (B) Expression of PfFtsHint in transformed AR423 cells was checked in lysates of bacteria grown at 30°C and 42 °C in a western blot probed with anti-GST antibody.

## Slide 6
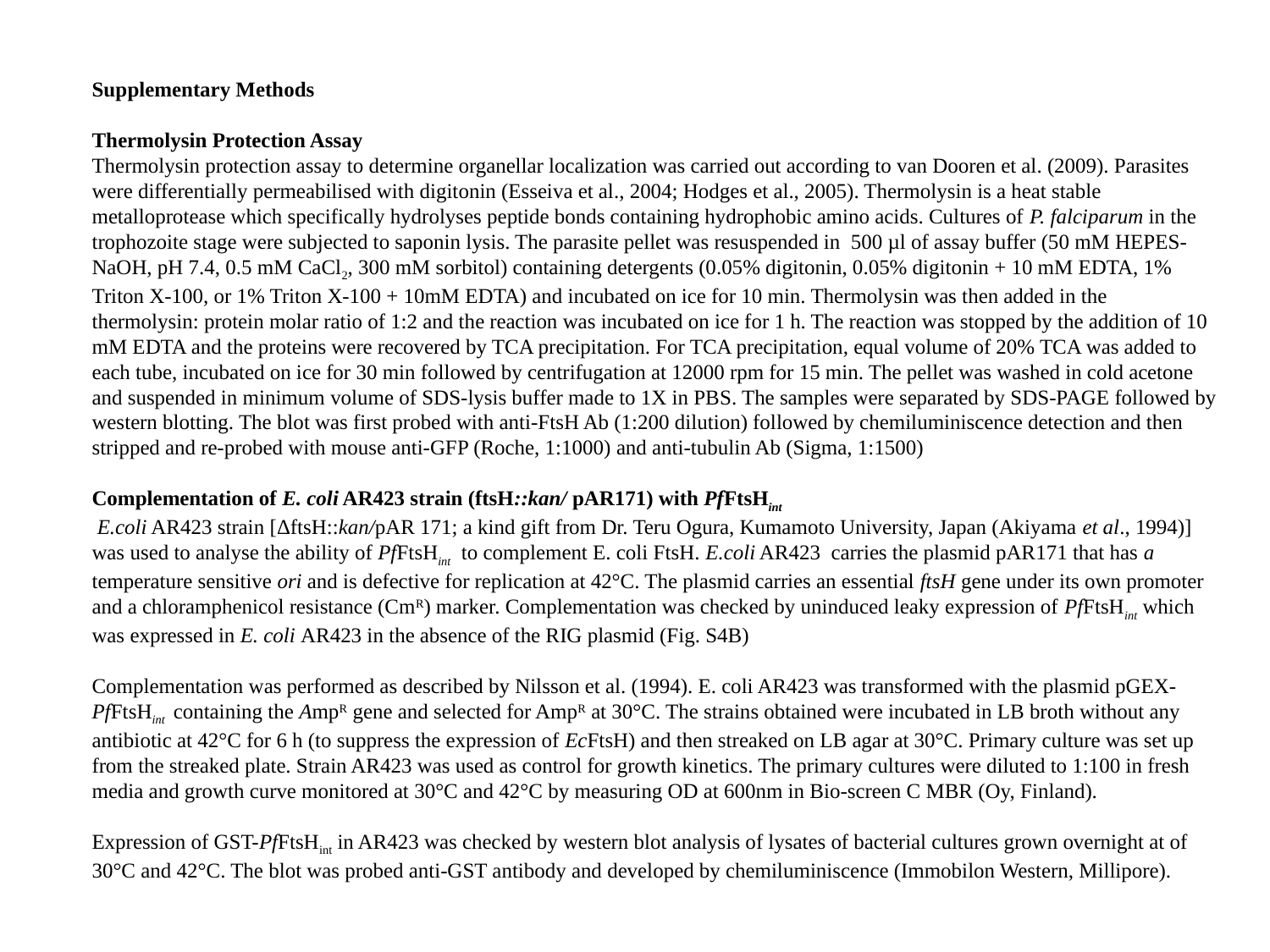

Supplementary Methods
Thermolysin Protection Assay
Thermolysin protection assay to determine organellar localization was carried out according to van Dooren et al. (2009). Parasites were differentially permeabilised with digitonin (Esseiva et al., 2004; Hodges et al., 2005). Thermolysin is a heat stable metalloprotease which specifically hydrolyses peptide bonds containing hydrophobic amino acids. Cultures of P. falciparum in the trophozoite stage were subjected to saponin lysis. The parasite pellet was resuspended in 500 µl of assay buffer (50 mM HEPES-NaOH, pH 7.4, 0.5 mM CaCl2, 300 mM sorbitol) containing detergents (0.05% digitonin, 0.05% digitonin + 10 mM EDTA, 1% Triton X-100, or 1% Triton X-100 + 10mM EDTA) and incubated on ice for 10 min. Thermolysin was then added in the thermolysin: protein molar ratio of 1:2 and the reaction was incubated on ice for 1 h. The reaction was stopped by the addition of 10 mM EDTA and the proteins were recovered by TCA precipitation. For TCA precipitation, equal volume of 20% TCA was added to each tube, incubated on ice for 30 min followed by centrifugation at 12000 rpm for 15 min. The pellet was washed in cold acetone and suspended in minimum volume of SDS-lysis buffer made to 1X in PBS. The samples were separated by SDS-PAGE followed by western blotting. The blot was first probed with anti-FtsH Ab (1:200 dilution) followed by chemiluminiscence detection and then stripped and re-probed with mouse anti-GFP (Roche, 1:1000) and anti-tubulin Ab (Sigma, 1:1500)
Complementation of E. coli AR423 strain (ftsH::kan/ pAR171) with PfFtsHint
 E.coli AR423 strain [ΔftsH::kan/pAR 171; a kind gift from Dr. Teru Ogura, Kumamoto University, Japan (Akiyama et al., 1994)] was used to analyse the ability of PfFtsHint to complement E. coli FtsH. E.coli AR423 carries the plasmid pAR171 that has a temperature sensitive ori and is defective for replication at 42°C. The plasmid carries an essential ftsH gene under its own promoter and a chloramphenicol resistance (CmR) marker. Complementation was checked by uninduced leaky expression of PfFtsHint which was expressed in E. coli AR423 in the absence of the RIG plasmid (Fig. S4B)
Complementation was performed as described by Nilsson et al. (1994). E. coli AR423 was transformed with the plasmid pGEX-PfFtsHint containing the AmpR gene and selected for AmpR at 30°C. The strains obtained were incubated in LB broth without any antibiotic at 42°C for 6 h (to suppress the expression of EcFtsH) and then streaked on LB agar at 30°C. Primary culture was set up from the streaked plate. Strain AR423 was used as control for growth kinetics. The primary cultures were diluted to 1:100 in fresh media and growth curve monitored at 30°C and 42°C by measuring OD at 600nm in Bio-screen C MBR (Oy, Finland).
Expression of GST-PfFtsHint in AR423 was checked by western blot analysis of lysates of bacterial cultures grown overnight at of 30°C and 42°C. The blot was probed anti-GST antibody and developed by chemiluminiscence (Immobilon Western, Millipore).

## Slide 7
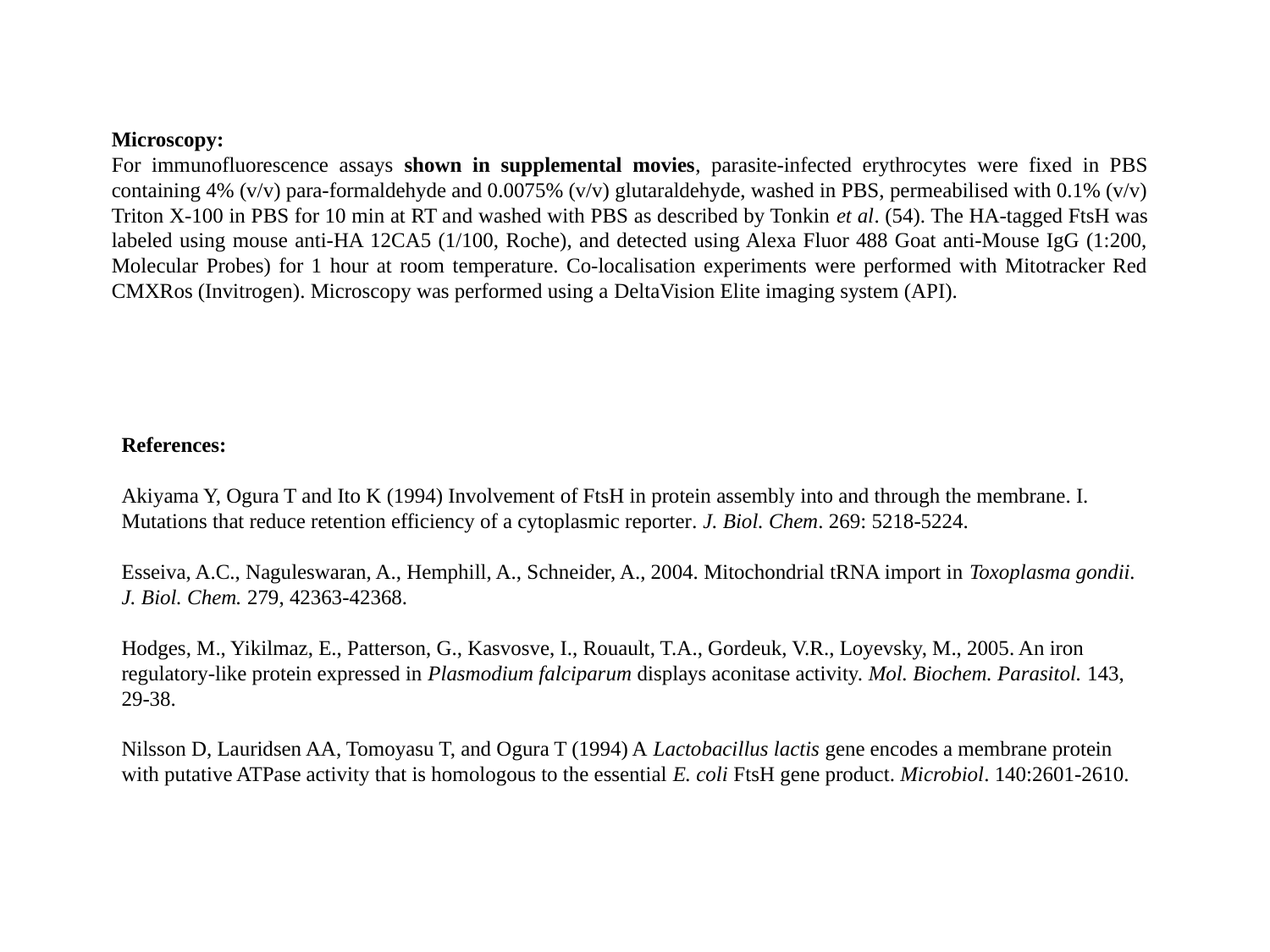

Microscopy:
For immunofluorescence assays shown in supplemental movies, parasite-infected erythrocytes were fixed in PBS containing 4% (v/v) para-formaldehyde and 0.0075% (v/v) glutaraldehyde, washed in PBS, permeabilised with 0.1% (v/v) Triton X-100 in PBS for 10 min at RT and washed with PBS as described by Tonkin et al. (54). The HA-tagged FtsH was labeled using mouse anti-HA 12CA5 (1/100, Roche), and detected using Alexa Fluor 488 Goat anti-Mouse IgG (1:200, Molecular Probes) for 1 hour at room temperature. Co-localisation experiments were performed with Mitotracker Red CMXRos (Invitrogen). Microscopy was performed using a DeltaVision Elite imaging system (API).
References:
Akiyama Y, Ogura T and Ito K (1994) Involvement of FtsH in protein assembly into and through the membrane. I. Mutations that reduce retention efficiency of a cytoplasmic reporter. J. Biol. Chem. 269: 5218-5224.
Esseiva, A.C., Naguleswaran, A., Hemphill, A., Schneider, A., 2004. Mitochondrial tRNA import in Toxoplasma gondii. J. Biol. Chem. 279, 42363-42368.
Hodges, M., Yikilmaz, E., Patterson, G., Kasvosve, I., Rouault, T.A., Gordeuk, V.R., Loyevsky, M., 2005. An iron regulatory-like protein expressed in Plasmodium falciparum displays aconitase activity. Mol. Biochem. Parasitol. 143, 29-38.
Nilsson D, Lauridsen AA, Tomoyasu T, and Ogura T (1994) A Lactobacillus lactis gene encodes a membrane protein with putative ATPase activity that is homologous to the essential E. coli FtsH gene product. Microbiol. 140:2601-2610.
