# Supplementary material for: An FtsH Protease Is Recruited to the Mitochondrion of Plasmodium falciparum
Source: PLoS One. 2013 Sep 13;8(9):e74408. doi: 10.1371/journal.pone.0074408 (PMC3772908; doi:10.1371/journal.pone.0074408)
Supplement: File S2 — (DOCX) [file pone.0074408.s002.docx]

>scer1_CAA56953

-PRDRIPIKYIERSS-PFTFLFPFLPTIILLGGLYFITRKINSSPPNFNKETDIKI--SFKNVAG-CDEAKQ

EIMEFVHFLKNPGKYTKLGAKIPRGAILSGPPGTGKTLLAKATAGEANVPFLSVSGSEFVEMFVGVGASRVR

DLFTQARSMAPSIIFIDEIDAIGKERGKGG-ALGGANDEREATLNQLLVEMDGFTTSD-------Q--VVVL

AGTNRPD-VLDNALMRPGRFDRHIQIDSPDVNGRQQIYLVHLKRLNLDPL------LSGKLATLTPGFTGAD

IANACNEAALIAARHNDP-YITIHHFEQAIERVIAGL-------EKK-TRVLSKE-EKRSVAYHEAGHAVCG

----WFLKYADPLLKVSIIPRGQGALGYAQYLPPD----QYLISEEQFRHRMIMALGGRVSEELHFPSVTSG

AHDDFKKVTQMANAMVTSLGMSPKIGYLSFDK-PFSNKTARTIDLEVKSIVDDAHRACTELLTKNLDKVDLV

AKELLRKEAITREDMIRLLGPR-

>scer2_CAA56955

-KDFRIPVLYVQEGN-WAKAMFQILPTVLMIAGIIWLTRRS------FNTETDVKI--KFKELAG-CDEAKE

EIMEFVSFLKEPSRYEKMGAKIPRGAILSGPPGTGKTLLAKATAGEAGVPFYFVSGSEFVEMFVGVGAARVR

DLFKTARENAPSIVFIDEIDAIGKARQKG--NFSGANDERENTLNQMLVEMDGFTPAD-------H--VVVL

AGTNRPD-ILDKALLRPGRFDRHINIDKPELEGRKAIFAVHLHHLKLA--------LKNRLAALTPGFSGAD

IANVCNEAALIAARSDED-AVKLNHFEQAIERVIGGV-------ERK-SKLLSPE-EKKVVAYHEAGHAVCG

----WYLKYADPLLKVSIIPRGQGALGYAQYLPGD----IFLLTEQQLKDRMTMSLGGRVSEELHFPSVTSG

ASDDFKKVTSMATAMVTELGMSDKIGWVNYQK-PFSDETGDIIDSEVYRIVQECHDRCTKLLKEKAEDVEKI

AQVLLKKEVLTREDMIDLLGKR-

>rglu_gi_342321438

-LGERIPVAYHDEIP-LGQTLLNLLPTLILIGSTVWIYRRMTGGGGGFNHETDIKV--GFRDVAG-MDEAKE

EIMEFVKFLKNPEHYERLGAKIPRGAILSGPPGTGKTLLAKATAGEAGVPFLSVSGSEFVEMFVGVGASRVR

DLFTQAKKNAPCIIFIDEIDAIGKARGKGG-QFGG-NDERESTLNQLLVEMDGFGTGE-------H--VVLL

AGTNRSD-VLDPALMRPGRFDRHIAVSNPDVSGRRQIFNVHLRKLVLDVPNSPLGKLIRKLAAHTPGFSGAD

IANVCNEAALIAARLGAD-VVKEKHFEMAIERVIAGM-------ERK-SRVLDKE-EKRTVAYHEAGHAVAG

----WFLEWADPLLKVSIVPRGVGALGYAQYLPKE----RYLYSTEQLIDRMCMTLGGRVAEEIFFKRITTG

AQDDLQKVTNMAMQTIANYGMNASVGPLSYRK-PFSEKTAQLIDDEVRKMVRDAHARCTQLLTEKRQQVEAV

AKVLLEKEVLSRHDMIEILGPR-

>hsap_CAB48398

-GENRVPVVYIAESD--GSFLLSMLPTVLIIAFLLYTIRRGPAG---LKDEIDVK----FKDVAG-CEEAKL

EIMEFVNFLKNPKQYQDLGAKIPKGAILTGPPGTGKTLLAKATAGEANVPFITVSGSEFLEMFVGVGPARVR

DLFALARKNAPCILFIDEIDAVGRKRGRG--NFGG-QSEQENTLNQLLVEMDGFNTTT-------N--VVIL

AGTNRPD-ILDPALLRPGRFDRQIFIGPPDIKGRASIFKVHLRPLKLDST------LARKLASLTPGFSGAD

VANVCNEAALIAARHLSD-SINQKHFEQAIERVIGGL-------EKK-TQVLQPE-EKKTVAYHEAGHAVAG

----WYLEHADPLLKVSIIPRGKG-LGYAQYLPKE----QYLYTKEQLLDRMCMTLGGRASEEIFFGRITTG

AQDDLRKVTQSAYAQIVQFGMNEKVGQISFDK-PYSEATARLIDDEVRILINDAYKRTVALLTEKKADVEKV

ALLLLEKEVLDKNDMVELLGPR-

>athal9_AAC33234

-RHEYVPVTYVSEMV-WYQEFMRFAPTLLLLGTLIYGARRMQGGLG-ADKHSKNK--IYFKDVAG-CDEAKQ

EIMEFVHFLKNPKKYEDLGAKIPKGALLVGPPGTGKTLLAKATAGESGVPFLSISGSDFMEMFVGVGPSRVR

HLFQEARQAAPSIIFIDEIDAIGRARGRG--GL-GGNDERESTLNQLLVEMDGFGTTA-------G--VVVL

AGTNRPD-ILDKALLRPGRFDRQITIDKPDIKGRDQIFKIYLKKIKLDHE----SYYSQRLAALTPGFAGAD

IANVCNEAALIAARHEGA-TVTMAHFESAIDRVIGGL-------EKK-NRVISKL-ERRTVAYHESGHAVVG

----WFLEHAEPLLKVTIVPRGTAALGFAQYVPNE----NLLMTKEQLFDMTCMTLGGRAAEQVLIGKISTG

AQNDLEKVTKMTYAQVAVYGFSDKVGLLSFPK-PYSNKTGAIIDEEVRDWVAKAYERTVELVEEHKVKVAEI

AELLLEKEVLHQDDLLKILGER-

>athal10_AAF79577

-SHDFVPVTYVSETI-WYQELLRFAPTLLLVATLIFGARRMQGGLGGADKNSKNK--IYFKDVAG-CEEAKQ

EIMEFVHFLQNPKKYEDLGAKIPKGALLVGPPGTGKTLLAKATAGESAVPFLSISGSDFMEMFVGVGPSRVR

NLFQEARQCAPSIIFIDEIDAIGRARGRG--GFSGGNDERESTLNQLLVEMDGFGTTA-------G--VVVL

AGTNRPD-ILDKALLRPGRFDRQITIDKPDIKGRDQIFQIYLKKIKLDHE----SYYSQRLAALTPGFAGAD

IANVCNEAALIAARHEGA-TVTMAHFDSAIDRVIGGL-------EKK-NRVISKL-ERRTVAYHESGHAVAG

----WFLEHAEPLLKVTIVPRGTAALGFAQYVPNE----NLLMTKEQLFDMTCMTLGGRAAEQVLIGRISTG

AQNDLEKVTKMTYAQVAVYGFSDKIGLLSFPK-PYSNRTGAMIDEEVREWVGKAYKRTVELIEEHKEQVAQI

AELLLEKEVLHQDDLTKVLGER-

>tpse1_gi_224004952

-PRDYVPVQYASETN-WAMELVKSAPALFLIGLTAYMLRGMGGMPG-IKKEDVS---VNFSDVAG-CQEAKK

EIMEFVEFLQDATQFTKLGAKIPKGALLTGPPGTGKTLLAKAVAGEANVPFYTISGSDFLEMFVGVGPSRVR

DLFKEARANAPCIVFIDEIDAVGRQRGRG--GFGGGNDERENTLNQLLVEMDGFSPST-------G--VVVL

AGTNRVD-ILDKALTRPGRFDRQITVDLPDLKGRKEVFLIHLKGIKLDGD----EDVAGRLAGLTPGFAGAD

IANICNEAAIVAARRKAE-SVTMDDFEKATDRIIGGL-------ES--NKIMSVD-EKSIVAHHEAGHAVAG

----WFLEHADPLLKVTIIPRSSGALGYAQYLPKE----VFLRTQEQIMDIVKMALAGRAAEEVFFGRVTTG

ASDDLRRVTQLVYSMIQVYGMNSRVGQLAFPK-PYSDATAEAMDEEARNIVDEAYSRTLELIRDKKTEVEAL

ANLLLEKETITHDDVIDAIGER-

>cmer1_BAA88164

-P---IPVVFRSENAGMFGELLRLLPTLIILGLGYALFRNSFSALS-IKKSAKGSERVTFAEVAG-LDEAKM

EVMELVDFLRDPKKYKDLGAKIPKGALLVGPPGTGKTLLAKAVAGEADVPFFSMSGSDFIEMFVGIRPSRVR

DLFAQARQNAPCIVFIDEIDAVGRARGRG--GFGGGNDERENTLNALLVEMDGFSSQE-------G--IVVL

AGTNRVD-ILDKALLRPGRFDRRINIDKPDIKGRFEIYKVHLRKIRIASS---VENVAKRLAALTPGFSGAD

IANSCNEAALIAARANKD-SVELADFESAIDRVIGGL-------EKK-NLVVLPE-EREIVAHHEAGHAVAS

----WFTKHADPLLKVSIVPRGSAALGFAQYLPRD----RFLQTREELEDFLVVALGGRAAEKLVFGRITTG

AQDDLERVTRLVYAAITRFGMSKRVGTISFNK-PFSEETAEIIDTEARTMVDKAYSRCEELLQAHLNELKAL

ARLLLEKEVVREDDLIQILGSK-

>hsap_CAA76314

-AKDRIPVSYKRTGF-FGNALYSVGMTAVGLAILWYVFRLAGMTGR-VDGKMGKG--VSFKDVAG-MHEAKL

EVREFVDYLKSPERFLQLGAKVPKGALLLGPPGCGKTLLAKAVATEAQVPFLAMAGPEFVEVIGGLGAARVR

SLFKEARARAPCIVYIDEIDAVGKKRSTT--MSGFSNTEEEQTLNQLLVEMDGMGTTD-------H--VIVL

ASTNRAD-ILDGALMRPGRLDRHVFIDLPTLQERREIFEQHLKSLKLTQS----TFYSQRLAELTPGFSGAD

IANICNEAALHAAREGHT-SVHTLNFEYAVERVLAGT-------AKK-SKILSKE-EQKVVAFHESGHALVG

----WMLEHTEAVMKVSITPRTNAALGFAQMLPRD----QHLFTKEQLFERMCMALGGRASEALSFNEVTSG

AQDDLRKVTRIAYSMVKQFGMAPGIGPISFPRRPFSQGLQQMMDHEARLLVAKAYRHTEKVLQDNLDKLQAL

ANALLEKEVINYEDIEALIGPP-

>pviv1_PVX_091615

-RDQLIEVQYTNEAN-VLHEVKSYIPTILFFLLFAFIFQKITLKN--KQHLKTDVR---FSNVAG-MKQAKE

EIMEFVDFLKTPSKYEALGAKMPKGALLCGAPGTGKTLLAKAVAGEANVPFFNISGSDFIEVFVGIGPSRVR

ELFAQARKHAPSIIFIDEIDAVGRKRSKGG-FAAGGNDERENTLNQMLVEMDGFHTSN-------DK-VVVL

AGTNRVD-ILDPAITRPGRFDRIVNISKPDINERSEIFQVHLKNLKLHHS----QSISYLLASLTPGFVGAD

IANVVNEGAIQCARRSNLVGVQVKDFELAIERVIGGL-------PK--SSSLISPFEKKIISYHETGHALIG

----WLLEFADPVLKVSILPRSNGALGYSQHLSEE----VFLFSREALLDKVAVILGGRAAEELFIGKITTG

AIDDLNKVTQLSYSYVSQYGMNKEIGLVSFQR-PHSECLAHLIDNEVRCLIETQYNRVKSILRTHEEQVHKL

ADLLFRKETISYQDIVQCIGER-

>pfal_PF11_0203

-RDELIEVQYVNEAN-ILNEVKGYIPSILFFLLLIFLFQKITLKN--KNSLKTDVK---FSSVAG-MKQAKE

EIMEFVDFLKNPTKYEILGAKIPKGALLCGAPGTGKTLLAKAVAGEANVPFFNISGSDFIEVFVGIGPSRVR

ELFAQARKHAPSIIFIDEIDAVGRKRSKGG-FSGGGNDERENTLNQMLVEMDGFHTSN-------DK-VVVL

AGTNRVD-ILDPAITRPGRFDRIVNISKPDINERSEIFQVHLKNLKLHES----KNISYILASLTPGFVGAD

IANVVNEGAIQCARRSNLLGVQIKDFELAIERVIGGL-------PK--SSSLISPLEKKIISYHETGHALIG

----WFLEFADPVLKVSIIPRNNGALGYSQHLSEE----IMLFSRDAILDKIAVILGGRAAEELFIGKITTG

AIDDLNKVTQLAYSYVSQYGMNQEIGLVSFQR-PHSECLAHLIDNEVRSLIETQYKRVKSILMKNEKHVHNL

ANLLYEKETISYHDIVKCVGER-

>bbov1_XP_001611654

-PQDYIGIHYVNEVN-VLGELKHYIPFMVVMMLLGLGLRKLTVR---AKDVKVDVK---FKDVAG-MHEAKK

EISEFVDFLKNPKAYEHYGAKIPKGALLCGAPGTGKTLLAKAVAGEANVPFYSISGSDFIEVFVGVGPSRVR

DLFEKARKNAPAIVFIDEIDAVGKKRAKGG-FSAGANDERENTLNQILVEMDGFKSSS-------G--VIVL

AGTNRAD-ILDPALVRPGRFDRTITINKPDLDERFEIFKVHLSPIKLNKN----DDVARRLAALTPSFVGAE

IANVSNEAAIQAVRRKSTDGVSLADFDAAIERVMAGL-------RR--SNALLSPAQKLAVAYHEVGHALIG

----WWLEHADPVLKVSIIPRSSGALGFSQQLPDE----AMLFSREALLDKVAVMLGGRAAEDIFIGRITTG

ATDDLNKVTRMCYAFVSQWGMNPALGLVSYQR-TYSENTAQLIDTEVRTMIESQYARVKSMLREKAELVHKL

SKLLYQRETITYHDIASCIGER-

>cpar_cgd1_3360

-TLNFVPIEYSHLIS-FKKIVNDLLPRAIGITIALLLLRSFSKS---VKNMNKNIK---FSDIAG-MKEAKQ

EIYELVEFLKDPKRFQDLGAKIPKGALLVGPPGTGKTLLAKAVAGEANVPFFYISGSDFIEIFVGMGASRVR

ELFSQARKLSPSIVFIDEIDAVGRKRAKGGGFAASSNDERESTLNQILVEMDGFTENN-------G--VIVL

AGTNRSD-VLDPALTRPGRFDRIINIERPNLEERKEIFKIHLKPLKLNEK----DELIKYLACLSPGFVGSE

IRNLCNEAAIHAARRTSNSGVDLIDFDKASDRIIGGL-------KK--LDGYLSPKEKKIVSLHESGHAIAG

----WYLKHADPILKVSIVPRTGGALGFAQMVPNE----LRLLSKEALLDKIAVLLAGRASEELYSESITTG

AYDDLQKATMIANSMITLYGMDPQIGLTTFNK-PYSEATSQAIDNCIRKMINDQYSRVKELLILKKEQVHKL

SDLLLNKETVTNQDINECIG---

>tgon1_TGME49_002630

LPRDFLPIYISDQHE-FH--LFDFLGSLFLFFLIANMVSELIFMRRMVKAETVKVR---FSDVAG-LHEAKR

EILEFVTFLKHPQSFRRLGAKLPKGALLVGPPGTGKTLLAKAVAGEAGVPFFSMSGSEFVEIFVGVGASRVR

ELFDEARKVAPSIIFIDEIDSVGAKRSTSF-----GNSERDNTLNQLLVEMDGFNPEE-------T--VVVL

AGTNRDD-LLDDALKRPGRFDRLVQIRRPDVAERKEIFKVHLKPLRLAPT----VALSERMAALTPGFVGAD

IANLCNEAAIQAARRRSKVGVEQRDFEAATERTIAGL-------PSP-VKDLLSSHQRRAIAYHECGHAIAG

----WFLKHGNPVLKLTIIPRSSGALGFAQQMPPT----VELHEKDALLDRIAVLLGGRAAEEIFIGAISSG

AADDIQKASRLARLSVMQFGMSDRLGLVDYSR-PYSEHTAKVIDDEVSQIINDQYERVKTLLKEREKEVHSL

CELLISRESITYSEILECIGPR-

>ncan1_NCLIV_022310

-PRDFLPIYISDHQE-FH--LFDFLGSLFLFVLIANMVSEVIFMRRMVKAETVKVR---FSDVAGELHEAKR

EILEFVTFLKHPQSFRRLGAKLPKGALLVGPPGTGKTLLAKVNS----FPLVSVGCR-----WRGRGAF---

-LLDERQRVR------------GDLRGR--------------------------------------------

--TNRDD-LLDDALKRPGRFDRLVQIRRPDVAERKEIFKVHLKPLRLAPT----VALAERMAALTPGFVGAD

IANLCNEAAIQAARRRSKLGVEQRDFEAATERTIAGL-------PSP-VKDLLSTYQRRAVAYHECGHAIAG

----WFLKHGNPVLKLTIIPRSSGALGFAQQMPPT----VELHEKDALLDRIAVLLGGRAAEEIFIGAVSSG

AADDIQKASRLARMSVMQFGMSDKLGLVDYSR-PYSEHTAKLIDEEVNNIINDQYERVKALLKEREKEVHKL

CELLINRESITYSEILECIGPR-

>tviv_gi_340055823

-EVVLRGTPVAEKGLVLFGTVAWVVPFVFFPVFVMLLSSSIITSLT-RVEHTSNTR---FHHIAG-MKEAKN

EVTEVVDFLRHPERYTALGAKVPTGAMLLGPPGTGKTLLAKAVAGESGVGFIPVCGSDFVELYVGMGALRVR

QLFEVAEKQR-CIVYIDEIDAIGLKRTGSG---FGEKQEQEHTLNELLTQLDGFSSRK-------RGDVMVL

ASSNVSQDKLDPALIRPGRFDRIIHVDMPVIAERIDIFKVHLSGLKLVSDRALINVYAERMSSLCPGFSGAD

IANVCNEAAILAARENCS-LVDITHLERSIDRVLAGI-------EHR-SRVLTPF-ERRVVAHHEAGHAVAG

----WFLNRADPLMKVSIVPRGGSALGYAQYLPNE----NNMRSAAEIRDSISVTLGGRVAEKIFFDHLSTG

ASDDLDKVARMAYMYVSSFSSRPVYPAPGTPK-PFGPAVSNEMDVEAKQLVDEIYESTYKLLLSKKKEMEIL

ANHLLESEVLTYDDVVRYLGVR-

>lmaj_gi_157873079

-ELTMKGRPFAETALIGVGAFAWVVPFIFFPVFVMFLSQYIGKSMS-RVETASNTR---FRDIAG-MKEPKK

EITEIVDFLRHPERYTKLGAKIPTGAMLLGPPGTGKTLLAKAVAGESGVGFIPAIGSDFVELYVGMGALRVR

QLFTEARKQR-CIIYIDEIDAIGLKRQGAG---HGEKQEQEQTLNELLTQLDGFSTGR-------RGDVMVL

ASSNVAQEALDPALIRPGRFDRIIHVDTPVISERTDIFKVHLSKLKLTPDRALIDAYAQRMSNLCPGFVGAD

IANVCNEGAILAAREGAD-HVSISHLERSIDRVLAGI-------EHR-SRALSDF-EKNVVAHHEAGHAVAG

----WFLHRADPLMKVSIVPRGGSALGYAQYLPNE----NFTRTAKEVRDSISVTLGGRAAEQIFFNHLSTG

ASDDLRKVAKMAYQYVSSFAPGSVYPPPGSNK-PFGVDKANDFDRRAKTLVDEVYADTLALLTKHKDDMKKL

ADHLLKHELLTYADVVHYLGER-

>tgon2_TGME49_059260

-DGADGVDIDSTDYEGVDDDGIRP-DGSYGNHMMHNTTDSSSGSNVTLVPPQPTFPPLTFNDLAG-LTEAKT

ELQEVVQFLRDPSKFERLGARLPKGVLLVGPPGTGKTALARAVATEAGVPYFYASGSEFVEIYVGQGARRVR

GLFSYARNHSPCIIFLDELDAVGGRRQASA-GPGAGNREHDQTLNQLLVEMDGFNQA---------SRIVVL

AATNRVD-TLDPALLRPGRFDRIVHVSLPDVAARELILQKYLQRV--PVETQVHKDLAKQIAKITPGFSGAE

LENLVNEAALLAARADKE-IVTLQELQEARDKVTMGP-------ARKT--RVMSAYQRQLTAYHEAGHAIIA

---FYLQPYADPIHKATIVSRGS-ALGFVEQVPLE---DRYGHGVAQLEARLCVCMGGRVAERFGRDALSNG

ASSDIETATRMAYVMVTEWGMSEKLGPLSYK-AFISSETANLVEEEVKQLVITAERKAEKLLRRHRKQLREV

ALQLLEKETLSGEEISEILDPSR

>ncan2_gi_401407042

-DGADSVDLDSTDYEGVDDEGIRPSDGGYGN-QMNNTTNSSLGSNAALVPPQPTFPPLTFNDLAG-LTEAKT

ELQEVVQFLRDPSKFERLGARLPKGVLLVGPPGTGKTALARAVATEAGVPYFYASGSEFVEIYVGQGARRVR

GLFSYARNHSPCIIFLDELDAVGGRRQAAG-GPGAGNREHDQTLNQLLVEMDGFNQA---------HRIVVL

AATNRVD-TLDPALLRPGRFDRIVHVSLPDVAARELILQKYLQRV--PVETQVHRDLAKQIAKITPGFSGAE

LENLVNEAALLAARADKD-AVTLQELHEARDKVTMGP-------ARKT--RIMSPYQRQLTAYHEAGHAILA

---FYLQPYADPIHKATIVSRGS-ALGFVEQVPLE---DRYGHGVAQLEARLCVCMGGRVAERFGRDALSNG

ASSDIETATRMAYVMVTEWGMSEKLGPLNYQ-AFISSETANLVEEEVKQLVMTAERKAEKLLRKHRRQLREV

ALQLLEKETLSGEEISDILDPSR

>pviv2_PVX_100935

-ITYKLFYKDNFNSSNFNTSNTSASLKPYGNDRSKKGEN-GKVSVKD-KNSSPHFRPIRFEEIAG-IDESKL

ELLEVVDFIRNREKYQEMGARMPKGVLLVGPPGSGKTMLARAVATEANVPYIYTSGPEFIEIYVGQGAKRIR

QLFAHARSVAPSIVFIDEIDAIGGKRSSGS-VNGAGQREHDQTLNQLLVEMDGFSNS---------IHIMVI

GATNRID-TLDSALLRPGRFDRIVYVPLPDVNGRKRILEIYIKKI--KSD---KAEDIDKIARLTPGFSGAD

LENVVNEATILATRNKKS-VVTIGELFEARDKVSMGP-------ERKS--LRQSDHQRRITAYHEAGHAIVA

---YFLQPKTDPIHKATIISRGN-ALGYVEQIPVD---DRHNYFKSQMEAKLAVCMGGRTAEEFGKSETSSG

ASSDISRATEIAYKMVTEWGMSDKLGPLNYKSNRLSAQTISTIEVEVKALVEKGKSLSEEILRRHRKELDNL

AFALLDRETLSGEEIKKIIDPNH

>pfal_PFL1925w

-ITYRLFYKDNFNNTNYRTNNSSTSLKSYGNEKNKKSDNNGKVPMKD-NKVSPHFKPIRFEEIAG-IDESKL

ELLEVVDFIKNREKYHEMGARMPKGVLLVGPPGSGKTMLARAVATEANVPYIYTSGPEFIEIYVGQGAKRIR

QLFAHARSVAPSIVFIDEIDAIGGKRSSGS-VNGAGQREHDQTLNQLLVEMDGFSNT---------VHIMVI

GATNRID-TLDSALLRPGRFDRIVYVPLPDINGRKKILEIYIKKI--KSD---KLEDIEKIARLTPGFSGAD

LENVVNEATILATRNNKS-LVTINELYEARDKVSMGP-------ERKS--LRQSDHQRRITAYHEAGHAIVA

---YFLQPKTDPIHKATIISRGN-ALGYVEQIPVD---DRHNYFKSQMEAKLAVCMGGRTAEEFGKSETSSG

ASSDISRATEIAYKMVTEWGMSDKLGPLNYKSNRLSAQTVSSIEVEVKSLVEKGKSLSEEILRRHRKELDNL

AFALLDKETLSGEEIKNIIDPND

>tpar_XP_766643

-IALGLFLTMFFFAMISHFLYNGNNINDFKNEPKKRSPPTPPPPKTQPPKPEVTFEPVHFKDILG-IDEAKE

DVQEIVKFIKQPFLYKKVGAKVPKGILLVGPPGTGKTMLAKAVATETGIPFIYTSGPEFVEIYVGQGAQRIR

ALFHKARKIAPCIIFIDEIDAVGSKRASGS-FSGQ-NREHDQTLNQLLVEMDGFNVS---------TGITIL

AATNRLS-ALDRALLRPGRFDRVVHIPLPSIKGREEILQHYLKDV--TYN---ETIDVKELSKITPGYSGAD

LKNLINEAALITVKQDRL-MVELSDLYEARDKIIMGN-------KRK---LLMPDIERKMTAYHEAGHALVA

---YYLYPNTDPIHKATIITRGT-ALGFVEQLPNDD-YDKSSYKLIEMKSRLAVCMAGRLAEKFGFDNVTSG

ASSDIIVATDLAYKMITQYGMSNKLASLNFH--KLSTDLNVKIENEIIELIKEAEHIAESILRSKRSQLELL

ASELLKYETLTGEQIKTLLKTN-

>bbov2_XP_001611107

-IAAGVVLTGLILTALGSFLVPKD--NDFGNVTPKNKPATRDDRTQQPERPPVMLERVYFKDIMG-IDEAKE

ELMEVVKFIKQPKLYHDIGAKIPKGVLLVGPPGTGKTMLAKAVATEANIPFIYTSGPEFVEIFVGQGAQRVR

NLFAKARKQAPCIVFIDEIDAIGAKRASGS-LGGQ-NREHDQTLNQLLVEMDGFNLS---------TGITVL

AATNRME-ALDRALLRPGRFDRVVHIPLPSLDGREAILKRYLSGI--KYD---DNVDVRALAKLTPGYSGAD

LKNLVNEAALNCVRSGRT-QVSTTDLQEARDKVGMGS-------IRR---TTQPELQRKMTAYHEAGHALVA

---FHLYPDADPVHKATIIHRGS-ALGFVEQLPED---DRQSYKLAQMKARLAICMGGRIAEEFGKENVTSG

ASSDIVAASELAYRMVTEWGMSPKLGPVNLRTRKLSHDTAQTVEQEVERLVSEAHFRAASILRRHRNQLERI

AERLLEEETLTGEQIRQIIAET-

>pviv3_PVX_117215

---VVDGSKKGMWGLLKSTIGF-LILVAAASVYLEGVSQNVQKGIGVKKIIPVENVKVTFADVKG-CDEVKQ

ELEEIIDYLKNSDKFTKIGAKLPKGILLSGEPGTGKTLIARAIAGEANVPFLQASGSEFEEMFVGVGARRIR

ELFQAAKKHAPCIVFIDEIDAVGSKRSN------RDNSAVRMTLNQLLVELDGFEQN---------EGIVVI

CATNFPQ-SLDKALVRPGRLDKTIVVPLPDIKGRYEILKMYSSKI--VLS----DVDLHVLSRRTVGMTGAD

LNNILNIAAIKCSVEGKK-AVDMNSIEQAFDRVVVGL-------QRK---SPLNEEEKNITAYHEGGHTLVN

---FYTK-GSDPVHKATIMPRGM-SLGVTWKIPIS---DKYSQKIRDVQSEIDILMGGLVSEEFGKNNVTTG

CSSDLQRATHIAQSLVMNYGINEENISMFLQ--NISEEMKIKIDKSIQRILLDSYNRAKKVLNQHIDELHRV

ASALVEYETLTSDEIKLAMQGK-

>pfal_PF14_0616

---VIDSNKKGLWNLLKSTIGF-LILVAAGSVYMEGVSQNVQKGIGVKKIIPVENVKVTFADVKG-CDEVKQ

ELEEIIDYLKNSDKFTKIGAKLPKGILLSGEPGTGKTLIARAIAGEANVPFLQASGSEFEEMFVGVGARRIR

ELFQAAKKHAPCIVFIDEIDAVGSKRSS------RDNSAVRMTLNQLLVELDGFEQN---------EGIVVI

CATNFPQ-SLDKALVRPGRLDKTIVVPLPDIKGRYEILKMYSNKI--VLS----DVDLHVLSRRTVGMTGAD

LNNILNIAAIKCSVEGKK-SVDMNSIEQAFDRVVVGL-------QRK---SPLNEEEKNITAYHEGGHTLVN

---FYTK-GSDPVHKATIMPRGM-SLGVTWKIPIS---DKYSQKIKDVQSEIDILMGGLVSEEFGKNNVTTG

CSSDLQKATHIAQSLVMNYGINEDNISMFLH--NISEEMKIKIDKSIQRILLDSYNRAKNVLNQHIDELHRI

ASALVEYETLTSDEIKLAMQGK-

>bbov3_XP_001609615

---VTPAASSKFMRFFKGLLSFGSIAFCFGSLYLL-LNQNLQRGLKHFKVVDPEDVDTTFADVKG-CDEVKR

ELDDVVDYLKNPEKFERLGAKLPKGILLSGPPGTGKTLLARAIAGEAGVPFIQASGSEFEEMFVGVGARRIR

ELFALARTMTPCIVFIDELDALGSKRSS------TDHNSVRMTLNQLLVELDGFSKR---------EGVVVL

CATNFPE-SLDPALVRPGRLDRTIHIPLPDYNGRYDILKLYSKKI--LVS----DVDLATIAKRTVGMTGAD

IFNILNMAALKCSIQGLA-SVTPSAIEEAFDRVVVGL-------KGK---PLTNERERKATAYHEGGHTLVS

---IHTP-GATQVHKATIAPRGR-TLGVTWKIPE----EKSDTRMSELHAEIAVLMGGMAAEEYGKENVSTG

CQSDLEKAADIARTMVMNFGMDDVSGPMFLDYAKLSEEHRKRVDTAVQKILNAGYRQASSVIRGNLVQLHNL

SDALVQYETLSADEIKHAIRGE-

>tgon3_TGME49_100020

-WRTFKVGSAGLWSLLLSPIAAVLLIWSFATEALPDLQQAAKKAEGLGKIVQPDQVKTTFNDVKG-CEEVKK

EVEEVVAYLKSPEKFTAMGARLPKGILLQGPPGTGKTLLARAIAGEAGVPFLHASGSEFEEMFVGVGASRLR

QLFAEARRLSPCILFIDEIDALGGKRTL------TENKHHRQTLNQLLTELDGFNPS---------DGVTLV

CATNLLE-ALDPALTRPGRIDRIIHVPFPSKKERIEILKHYAKEM--PLA----DVDLEALAGLTSGMTGAD

LSNLLNFAAIRAATEGKE-QVTRAEVDESFDRLMVGS-------RRTG--VVMKEEERRLTAYHESGHALVA

---LYTP-ASAPLHKATILFRGS-SLGVTWSVEKE---DTFSQSEQQCLASLDVAMGGKAAEEFGAGKVTSG

CRSDLVRATQLARAMVTNYGFTDSKAPMVIGYLLVSDEKKSRVDEAVQKLLDESYARARRLLEEKRDELTRL

AEALLEHETLSAEEVRLAVAGKR

>ncan3_NCLIV_064680

-WKTVKVGSRGLWSLLLSPVAAVLLIWSFATEALPGLQQAAKKVDGLGKIVQPDEVKTTFSDVKG-CEEVKK

EVEEVVAYLKSPAKFTAMGARLPKGILLQGPPGTGKTLLARAIAGEAGVPFIHASGSEFEEMFVGVGASRLR

QLFAEARRLSPCILFIDEIDALGGKRTL------TENKHHRQTLNQLLTELDGFKPS---------DGVTLV

CATNLLE-ALDPALTRPGRIDRIIHVPFPSKKERIEILQHYAKEM--PLA----DVDLEILAALTSGMTGAD

LANLLNFAAIRAATEGKE-QVTRAEVDESFDRLMVGS-------RRAG--VVMKEEERRLTAYHESGHALVA

---LYTP-ASAPLHKATILFRGS-SLGVTWSVEKE---DSYSQSEQQCLASLDVAMGGKAAEAFGEGNVTSG

CRSDLVRATQLARTMVTNYGFSDSKAPMVIGYAVVSDERRSRIDEAVQKLLDESYARARKLLEEKREELKRL

AEALLEHETLSAEEVRLAVSGKD

>athal11_AAC31223

-STERTHFKEQLWSTIR-TIGVG-FLLISGIGALIEDRGIGK---GLEEVQPSMDSSTKFSDVKG-VDEAKA

ELEEIVHYLRDPKRFTRLGGKLPKGVLLVGPPGTGKTMLARAIAGEAGVPFFSCSGSEFEEMFVGVGARRVR

DLFSAAKKCSPCIIFIDEIDAIGGSRNP------KDQQYMKMTLNQMLVELDGFKQN---------EGIIVV

AATNFPE-SLDKALVRPGRFDRHIVVPNPDVEGRRQILESHMSKV--LKA----DVDLMIIARGTPGFSGAD

LANLVNVAALKAAMDGSK-DVTMSDLEFAKDRIMMGS-------ERKS--AVISDESRKLTAFHEGGHALVA

---IHTE-GALPVHKATIVPRGM-ALGMVSQLPDK---DETSISRKQMLARLDVCMGGRVAEEFGESEVTSG

ASSDLEQATKLARAMVTKF-MSKEVGLVAHNGKSMSTETRLLIESEVKQLLEKAYNNAKTILTVYNKELHAL

ANALLQHETLSGKQIKELLTDL-

>tpse2_gi_224015872

----------------------------------------------MKHIQEAEGSDVRFSDVKG-VTEAKA

ELEEIVLYLKDPERFTRLGGKLPRGLLLTGPPGTGKTLLAKAIAGEAGVPFFFSSGSQFEEVYVGLGAKRIR

ELFEAAKQKSPSIIFIDEIDAVGGTRKL------KDQSALKMTLNELLVQMDGFDEN---------NGIIVI

GATNFAE-SLDSALLRPGRFDKSVVVPLPDVGGRKEILEMYAAKT--KVS----DVDLGILARGTTGFSGAD

LYNLMNQAALKASIDGLD-NITMQIFEWAKDKIIMGA-------ERKS--AVITPETAKCTAYHEAGHALVG

---VLTD-GARTIHKATIMPRGQ-ALGMVTTLPEG---DETSMSLKQMIAMMDVCMGGRVAEEFGEENVTSG

ASSDIQYATRIARSMVTKY-FSDDVGIVYYGQDDASGKTRSQIDDEVKRLTSAAYDRAKNLLKKHSREHKLL

AETLLEYETLTGDEVRELILEG-

>athal12_BAB08420

-VSNKSRFAQELVSTILFTVAVG-LVWIMGAAALQKYIGSLG---GIKEITPEKNVKT-FKDVKG-CDDAKQ

ELEEVVEYLKNPSKFTRLGGKLPKGILLTGAPGTGKTLLAKAIAGEAGVPFFYRAGSEFEEMFVGVGARRVR

SLFQAAKKKAPCIIFIDEIDAVGSTRKQ------WEG-HTKKTLHQLLVEMDGFEQN---------EGIIVM

AATNLPD-ILDPALTRPGRFDRHIVVPSPDVRGREEILELYLQGK--PMS----DVDVKAIARGTPGFNGAD

LANLVNIAAIKAAVEGAE-KLSSEQLEFAKDRIVMGT-------ERKT--MFVSEDSKKLTAYHESGHAIVA

---LNTK-GAHPIHKATIMPRGS-ALGMVTQLPSN---DETSVSKRQLLARLDVCMGGRVAEEFGLDHITTG

ASSDLSQATELAQYMVSSC-MSEAIGPVHIK-ERPSSDMQSRIDAEVVKLLREAYERVKSLLKRHEKQLHTL

ANALLEYETLTAEDIKRILLPK-

>scer3_AAA02883

-VSESTFTVVSRWVKWLLVFGILTYSFSEGFKYITENTTLLKSSE--DKSVDVAKTNVKFDDVCG-CDEARA

ELEEIVDFLKDPTKYESLGGKLPKGVLLTGPPGTGKTLLARATAGEAGVDFFFMSGSEFDEVYVGVGAKRIR

DLFAQARSRAPAIIFIDELDAIGGKRNP------KDQAYAKQTLNQLLVELDGFSQT---------SGIIII

GATNFPE-ALDKALTRPGRFDKVVNVDLPDVRGRADILKHHMKKI--TLA----NVDPTIIARGTPGLSGAE

LANLVNQAAVYACQKNAV-SVDMSHFEWAKDKILMGA-------ERKT--MVLTDAARKATAFHEAGHAIMA

---KYTN-GATPLYKATILPRGR-ALGITFQLPEM---DKVDITKRECQARLDVCMGGKIAEEYGKDNTTSG

CGSDLQSATGTARAMVTQY-MSDDVGPVNLSWESWSNKIRDIADNEVIELLKDSEERARRLLTKKNVELHRL

AQGLIEYETLDAHEIEQVCKGE-

>mmus_AAC35558

-FLKAQALTQKTNDSLRRTRLILFVLLLFGIYGLLKNPFLSVRFRTTSAVDPVQMKNVTFEHVKG-VEEAKQ

ELQEVVEFLKNPQKFTVLGGKLPKGILLVGPPGTGKTLLARAVAGEADVPFYYASGSEFDEMFVGVGASRIR

NLFREAKANAPCVIFIDELDSVGGKRIES-----PMHPYSRQTINQLLAEMDGFKPN---------EGVIII

GATNFPE-ALDNALIRPGRFDMQVTVPRPDVKGRTEILKWYLNKI--KFD----SVDPEIIARGTVGFSGAE

LENLVNQAALKAAVDGKE-MVTMKELEFSKDKILMGP-------ERRS--VEIDNKNKTITAYHESGHAIIA

---YYTK-DAMPINKATIMPRGP-TLGHVSLLPEN---DRWNETRAQLLAQMDVSMGGRVAEEFGTDHITTG

ASSDFDNATKIAKRMVTKF-MSEKLGVMTYSTGKLSPETQSAIEQEIRILLRESYERAKHILKTHAKEHKNL

AEALLTYETLDAKEIQIVLEGK-

>hsap_CAB51858

-FLKAQALTQKTNDSLRRTRLILFVLLLFGIYGLLKNPFLSVRFRTTSAVDPVQMKNVTFEHVKG-VEEAKQ

ELQEVVEFLKNPQKFTILGGKLPKGILLVGPPGTGKTLLARAVAGEADVPFYYASGSEFDEMFVGVGASRIR

NLFREAKANAPCVIFIDELDSVGGKRIES-----PMHPYSRQTINQLLAEMDGFKPN---------EGVIII

GATNFPE-ALDNALIRPGRFDMQVTVPRPDVKGRTEILKWYLNKI--KFD----SVDPEIIARGTVGFSGAE

LENLVNQAALKAAVDGKE-MVTMKELEFSKDKILMGP-------ERRS--VEIDNKNKTITAYHESGHAIIA

---YYTK-DAMPINKATIMPRGP-TLGHVSLLPEN---DRWNETRAQLLAQMDVSMGGRVAEEFGTDHITTG

ASSDFDNATKIAKRMVTKF-MSEKLGVMTYSTGKLSPETQSAIEQEIRILLRDSYERAKHILKTHAKEHKNL

AEALLTYETLDAKEIQIVLEGK-

>athal1_CAB61952

-GNNVEFGSPEKRSGGFFNSALIALFYIAVLAGLIR-FPVSFSTS--GKVS-GGGETITFADVAG-VDEAKE

ELEEIVEFLRNPEKYVRLGARPPRGVLLVGLPGTGKTLLAKAVAGEAEVPFISCSASEFVELYVGMGASRVR

DLFARAKKEAPSIIFIDEIDAVAKSRDGKF-RM-GSNDEREQTLNQLLTEMDGFDSN---------SAVIVL

GATNRAD-VLDPALRRPGRFDRVVTVETPDKIGRESILRVHVSKKELPLG-----VNLGSIASMTTGFTGAD

LANLVNEAALLAGRKNKT-NVEKIDFIQAVERSIAGI-------EKK--SARLKGNEKAVVARHEAGHAVVG

TAVANLLTGQPRVEKLSILPRTGGALGFTYIPPTS--EDRYLLFIDELLGRLVTLLGGRAAEEYSGR-ISTG

AFDDIRRATDMAYKAVAEYGLNQKIGPVSVA-GRDQGKLVDLVQKEVTILLQSALDVALSVVRANPDVLEGL

GAQLEEKEKVEGEELQKWLSMV-

>athal2_BAB09632

-ENNVEFGSPDKRSGGFFNSGLIVLFYIAVLAGLLHRFPVNFSQS--GKVS-GDGETITFADVAG-VDEAKE

ELEEIVEFLKNPDRYVRLGARPPRGVLLVGLPGTGKTLLAKAVAGESDVPFISCSASEFVELYVGMGASRVR

DLFARAKKEAPSIIFIDEIDAVAKSRDGKF-RM-VSNDEREQTLNQLLTEMDGFDSS---------SAVIVL

GATNRAD-VLDPALRRPGRFDRVVTVESPDKVGRESILKVHVSKKELPLG-----VNLASIASMTTGFTGAD

LANLVNEAALLAGRKSKM-TVDKIDFIHAVERSIAGI-------EKK--TARLKGSEKAVVARHEAGHAVVG

TAVASLLSGQSRVEKLSILPRSGGALGFTYIPPTH--EDRYLLFIDELHGRLVTLLGGRAAEEYSGR-ISTG

ALDDIRRATDMAYKAVAEYGLNEKIGPVSVA-GRDQGHLVDLVQREVTNLLQSALDVALTVVRANPDVLEGL

GAQLEDEEKVEGEELQKWLNRV-

>syne1_BAA17477

-AKGIEFAAAPPAKNSWFGTLLSWVIPPLIFVGIWSFFLNRNNNG--VYVE-GDSTKVTFDDVAG-VEEAKT

ELSEVVDFLKFPQRYTALGAKIPKGVLLVGPPGTGKTLLAKAAAGEAGVPFFIISGSEFVELFVGAGAARVR

DLFEQAKKQAPCIVFIDELDAIGKSRASGA-FM-GGNDEREQTLNQLLTEMDGFSAAG--------ATVIVL

AATNRPE-TLDPALLRPGRFDRQVLVDRPDLAGRLKILEIYAKK--IKLD-----VELKNIATRTPGFAGAD

LANLVNEAALLAARNKQD-SVTEADFREAIERVVAGL-------EKK--SRVLSDKEKKIVAYHEVGHALVG

----AVMPGGGQVAKISIVPRGMAALGYTLQMPT---EDRFLLNESELRDQIATLLGGRAAEEFDS--ITTG

AANDLQRATDLAEQMVTTYGMSKVLGPLAYD-RMVSDDTAKEIDLEVKEIVEQGHNQALAILEHNRDLLEAI

AEKILEKEVIEGEELHHLLGQV-

>athal3_AAD50055

-ISVSE---GESSGNDLFTVIGNLIFPLLAFGGLFLLFRRAQGGP-GKFQE-VPETGVSFADVAG-ADQAKL

ELQEVVDFLKNPDKYTALGAKIPKGCLLVGPPGTGKTLLARAVAGEAGVPFFSCAASEFVELFVGVGASRVR

DLFEKAKSKAPCIVFIDEIDAVGRQR-GAG-MG-GGNDEREQTINQLLTEMDGFSGN---------SGVIVL

AATNRPD-VLDSALLRPGRFDRQVTVDRPDVAGRVKILQVHSRGK--ALG-----VDFDKVARRTPGFTGAD

LQNLMNEAAILAARRELK-EISKDEISDALERIIAGP-------EKK--NAVVSEEKKRLVAYHEAGHALVG

----ALMPEYDPVAKISIIPRGQ-AGGLTFFAPSEERLESGLYSRSYLENQMAVALGGRVAEEFGDENVTTG

ASNDFMQVSRVARQMIERFGFSKKIGQVAVG-KDYSMATADIVDAEVRELVEKAYKRATEIITTHIDILHKL

AQLLIEKETVDGEEFMSLFIDG-

>athal4_BAB10200

-ISVSE---GEG-GNGLFDFIGNLLFPLLAFGGLFYLFRGGQGG--AKFQE-VPETGVTFGDVAG-ADQAKL

ELQEVVDFLKNPDKYTALGAKIPKGCLLVGPPGTGKTLLARAVAGEAGVPFFSCAASEFVELFVGVGASRVR

DLFEKAKSKAPCIVFIDEIDAVGRQR-GAG-MG-GGNDEREQTINQLLTEMDGFSGN---------SGVIVL

AATNRPD-VLDSALLRPGRFDRQVTVDRPDVAGRVQILKVHSRGK--AIG-----VDYEKVARRTPGFTGAD

LQNLMNEAAILAARRELK-EISKDEISDALERIIAGP-------EKK--NAVVSEEKKRLVAYHEAGHALVG

----ALMPEYDPVAKISIIPRGQ-AGGLTFFAPSEERLESGLYSRSYLENQMAVALGGRVAEEFGDENVTTG

ASNDFMQVSRVARQMVERFGFSKKIGQVAVG-KDYSMATADVVDAEVRELVEKAYVRAKEIITTQIDILHKL

AQLLIEKETVDGEEFMSLFIDG-

>ntab_BAA33755

-ISVSE---GDSAGNGLFNLIGN-LFPFIAFAGLFYLFQRSQGG--PKFQE-VPETGVTFADVAG-ADQAKL

ELQEVVDFLKNPDKYTALGAKIPKGCLLVGPPGTGKTLLARAVAGEAGVPFFSCAASEFVELFVGVGASRVR

DLFEKAKSKAPCIVFIDEIDAVGRQR-GAG-MG-GGNDEREQTINQLLTEMDGFSGN---------SGVIVL

AATNRPD-VLDSALLRPGRFDRQVTVDRPDVAGRIKILQVHSRGK--ALT-----VDFEKIARRTPGYTGAD

LQNLMNEAAILAARRELK-EISKDEISDALERIIAGP-------EKK--NAVVSDEKKKLVAYHEAGHALVG

----ALMPEYDPVAKISIIPRGQ-AGGLTFFAPSEERLESGLYSRSYLENQMAVALGERVAEEFGQDNVTTG

ASNDFMQVSRVARQMVERLGFSKKIGQVAIG-KDYSMATADVVDAEVRELVERAYERATEIITTHIDILHKL

AQLLIEKETVDGEEFMSLFIDG-

>syne2_BAA17010

-IAVQ----PQSDEGFWFRIASTLFLPILLLVGIFFLFRRAQSG---RVQM-EPQTQVTFGDVAG-IEQAKL

ELTEVVDFLKNADRFTELGAKIPKGVLLVGPPGTGKTLLAKAVAGEAGVPFFSISGSEFVEMFVGVGASRVR

DLFEQAKANAPCIVFIDEIDAVGRQR-GAG-LG-GGNDEREQTLNQLLTEMDGFEGN---------TGIIIV

AATNRPD-VLDSALMRPGRFDRQVVVDRPDYAGRREILNVHARGK--TLS-----VDLDKIARRTPGFTGAD

LSNLLNEAAILAARRNLT-EISMDEVNDAIDRVLAGP-------EKK--NRVMSEKRKTLVAYHEAGHALVG

----ALMPDYDPVQKISIIPRGR-AGGLTWFTPSEDRMESGLYSRSYLQNQMAVALGGRIAEEFGEEEVTTG

ASNDLQQVARVARQMVTRFGMSDRLGPVALG-RDFSDETAAAIDEEVSQLVDQAYQRAKQVLVENRGILDQL

AEILVEKETVDSEELQTLLANN-

>tpse3_gi_224003627

-ISVSSFANLPAQRNFIASFLKRLLFPLSIFAGLFFLLKRSAGSS--SFNF-HPTTNITFEDVAG-CDGAKL

ELAEIVDFLKQPQAYTNNGCRIPAGALLYGPPGTGKTLLAKAVAGEAGVPFVSMSGSEFVELYVGVGASRVR

ELFFQAKKNAPCIVFLDEIDAVGRQR-GAG-YA-GGNDEREQTINQILVEMDGFDGN---------IGVITL

AATNRLD-ILDEALLRPGRFDRKISVDLPDVHGRTKILSVHSRGK--PLE-----VDLDAIARRTPGFSGAE

LENLMNEAALSAARQGKE-TIGWMEVDGALDRLMVGM-------EKSGGTSYLSQKQKELVAYHEAGHAICG

----ALIPDYDQVQKISIIPRSNGAGGLTFFSPQEARLESGMYSKQYLESQLVVALGGRVAEEFGEDSVTTG

ASNDLDHVSSIAKQMVKEWGMSNVVGPLALSRKVWGPKMMGLVDGEVERLVNNAYVNAKHILTENKDLLEHL

AYTLVEQESVSAEEFQFMLL---

>tpse4_gi_223995685

-VTVLPSNEAAGG---LGDLAQSLILPAALFAGLFFLSRRAGGGAGMQIQM-IPDTGVNFEDVAG-CDGAKL

ELAEVVDFLKQPEVYSKNGCRIPRGVILDGPPGTGKTLLAKAVAGEAGVPFISISGSEFVEMFVGVGASRVR

DVFSQAKKNAPCIIFIDEIDAVGRQR-GAG-FA-GGNDEREQTINQILVEMDGFDGN---------PGIITI

AATNRVD-ILDQALLRPGRFDRKITVDLPDFKGRTRILGVHARGK--PLE-----VDLEAIGRRTPGFSGAQ

LENLMNEAAISAARIGKS-TIGWEQIDGAVDRIMVGL-------EKKGGTAMLSAKQNELVAYHEAGHAICG

----ALIPDYDQVQKISIIPRSNGAGGLTFFAPQEQRLESGMYSKQYLESQLAVALGGRLAEEYGEDFVTTG

ASNDIQQVANIAKRMVKEWGMSEIVGPIALSQTTWGGKILSNVDGEVERLVNNSYITAKHILSENMDLLHHL

AKTLVEQEVVSAEEFQMMLVE--

>athal5_AAF24819

-IDFAAHNAQEDQGSPILNLIGNLAFPVILIGGLFLLSRRSSGGM--KFQM-EPNTGVTFDDVAG-VDEAKQ

DFMEVVEFLKKPERFTAVGARIPKGVLLVGPPGTGKTLLAKAIAGEAGVPFFSISGSEFVEMFVGVGASRVR

DLFKKAKENAPCIVFVDEIDAVGRQR-GTG-IG-GGNDEREQTLNQLLTEMDGFEGN---------TGVIVV

AATNRAD-ILDSALLRPGRFDRQVSVDVPDVKGRTDILKVHSGNK--KFE-----VSLEVIAMRTPGFSGAD

LANLLNEAAILAGRRGKT-AISSKEIDDSIDRIVAGM-------EGT---VMTDGKSKSLVAYHEVGHAICG

----TLTPGHDAVQKVTLIPRGQ-ARGLTWFIPSD---DPTLISKQQLFARIVGGLGGRAAEEFGESEVTTG

AVSDLQQITGLAKQMVTTFGMS-EIGPWSLM-NSMSEKLANDIDTAVKTLSDKAYEIALSQIRNNREAMDKI

VEILLEKETMSGDEFRAILSEF-

>athal6_AAF65925

-IDFAAHNAQEDQGSVLFNLIGNLAFPALLIGGLFLLSRRSGGGM--KFQM-EPNTGVTFDDVAG-VDEAKQ

DFMEVVEFLKKPERFTAVGAKIPKGVLLIGPPGTGKTLLAKAIAGEAGVPFFSISGSEFVEMFVGVGASRVR

DLFKKAKENAPCIVFVDEIDAVGRQR-GTG-IG-GGNDEREQTLNQLLTEMDGFEGN---------TGVIVV

AATNRAD-ILDSALLRPGRFDRQVSVDVPDVKGRTDILKVHAGNK--KFD-----VSLEIIAMRTPGFSGAD

LANLLNEAAILAGRRART-SISSKEIDDSIDRIVAGM-------EGT---VMTDGKSKSLVAYHEVGHAVCG

----TLTPGHDAVQKVTLIPRGQ-ARGLTWFIPSD---DPTLISKQQLFARIVGGLGGRAAEEFGDSEVTTG

AVGDLQQITGLARQMVTTFGMS-DIGPWSLM-NSMSEKLAEDIDSAVKKLSDSAYEIALSHIKNNREAMDKL

VEVLLEKETIGGDEFRAILSEF-

>ntab_AAD17230

-IDFAAHNAQEDSGSFLFNLIGNLAFPLILIGGLFLLSRRSPGGM--KFQM-EPNTGVTFDDVAG-VDEAKQ

DFMEVVEFLKKPERFTAVGARIPKGVLLVGPPGTGKTLLAKAIAGEAGVPFFSISGSEFVEMFVGVGASRVR

DLFKKAKENAPCIVFVDEIDAVGRQR-GTG-IG-GGNDEREQTLNQLLTEMDGFEGN---------TGIIVV

AATNRAD-ILDSALLRPGRFDRQVSVDVPDIKGRTEILKVHAGNK--KFD-----VSLEVIAMRTPGFSGAD

LANLLNEAAILAGRRGKT-AIASKEIDDSIDRIVAGM-------EGT---VMTDGKSKSLVAYHEVGHAICG

----TLTPGHDAVQKVTLIPRGQ-AKGLTWFIPAD---DPTLISKQQLFARIVGGLGGRAAEEFGEPEVTTG

AAGDLQQITGLAKQMVVTFGMS-ELGPWSLM-NSMSEKLAEDIDGAVKRLSDSAYEIALTHIRNNREAIDKI

VEVLLEKETMTGDEFRAILSEF-

>athal7_CAB89335

-VDFAAHPMNVNWGAFLLNFLGNLGFPLILLVSLLLTSSSRRN----KFQM-EPNTGITFEDVAG-VDEAKQ

DFEEIVEFLKTPEKFSALGAKIPKGVLLTGPPGTGKTLLAKAIAGEAGVPFFSLSGSEFIEMFVGVGASRAR

DLFNKAKANSPCIVFIDEIDAVGRMR-GTG-IG-GGNDEREQTLNQILTEMDGFAGN---------TGVIVI

AATNRPE-ILDSALLRPGRFDRQVSVGLPDIRGREEILKVHSRSK--KLD-----VSLSVIAMRTPGFSGAD

LANLMNEAAILAGRRGKD-KITLTEIDDSIDRIVAGM-------EGT---KMIDGKSKAIVAYHEVGHAICA

----TLTEGHDPVQKVTLVPRGQ-ARGLTWFLPGE---DPTLVSKQQLFARIVGGLGGRAAEDFGEPEITTG

AAGDLQQVTEIARQMVTMFGMS-EIGPWALT-NSMSEKLAEDIDSCVKKIIGDAYEVAKKHVRNNREAIDKL

VDVLLEKETLTGDEFRAILSEY-

>tpse5_gi_118411141

-IDFDAHPAEQK--NLFVTIASNLLLPIIFIAGLVYFFQNSENFG--RFER-RPDTGVNFNDIAG-IDEAKA

EFEEIVSFLKEPEKYTVVGAKIPKGILLVGPPGTGKTLLAKAIANEADVPFFSVAGSEFVEMFIGIGAARVR

DLFQKASENAPCIVFIDEIDAVGRER-GAG-VG-GGNDEREQTLNQLLTEMDGFKEN---------KGVIVV

GATNRVD-ILDAALLRPGRFDRQVTVNLPDRLGRISILKVHAKNK--PLG-----VSLVQLANRTPGFSGAD

LANLLNEAAILATRYKKE-TISKNEVNQAIDRIIGGI-------AGT---PMEDSKNKKLIAYHEVGHAITG

----TVLQSHDEVEKITITPRGN-AKGLTWFTPEE---DQSLISRSALLARIIGTLGGRAAEQFGDPEVTTG

ASSDLQQVTNLARQMVTRFGMS-NIGPIALE-SGYPESIADRIDDEVCKIISYCEQKALQIILDNRVIIDLI

VERLLDLETMEGDEFRELLSSY-

>cmer2_BAA88165

-IDVDVHAVSN-----WINVASNWIIPLIIIGVVIWLLSRSASS---RFQM-VAKTGIMFDDVAG-IEEAKE

ELAEVVAFLKNPSKFLAVGASIPKGVLLVGPPGTGKTLLAKAIAGEASVPFFSISGSEFVEMFVGVGASRVR

DLFKKAKQNAPCLVFIDEIDAVGRQR-GAG-IG-GGNDEREQTLNQLLTEMDGFEGN---------TGVIVI

AATNRVD-VLDAALLRPGRFDRQIMVSMPDVKSRIAILKVHANQK--KLH-----VSLEAVARRTAGFAGAD

LANLLNEAAILAVRRGLK-QITWKEIDDAIDRVIAGM-------EGT---PIMDGKIKRLIAYHETGHALTA

----TLLPNHPPVQKVTLIPRRQ-AKGLTWFMQDN---ERDLLSKSQLMSMIMVALGGRAAEEFGNAEVTTG

ASNDLQQVTNLARQMVTRFGMS-SLGPLCLE-PEVSEEVIAQIDAQVRGMIEACYEKVLELMQANRVVMDRI

VEELMEKETLDGKEFRQLVSQA-

>syne3_BAA10230

-IRLDSHPVRNN--GMVWGFVGNLIFPVLLIASLFFLFRRSSNMP--RFQM-DAKTGVMFDDVAG-IDEAKE

ELQEVVTFLKQPERFTAVGAKIPKGVLLVGPPGTGKTLLAKAIAGEAGVPFFSISGSEFVEMFVGVGASRVR

DLFKKAKENAPCLIFIDEIDAVGRQR-GAG-IG-GGNDEREQTLNQLLTEMDGFEGN---------TGIIII

AATNRPD-VLDSALMRPGRFDRQVMVDAPDYSGRKEILEVHARNK--KLA-----VSIDSIARRTPGFSGAD

LANLLNEAAILTARRRKS-AITLLEIDDAVDRVVAGM-------EGT---PLVDSKSKRLIAYHEVGHAIVG

----TLLKDHDPVQKVTLIPRGQ-AQGLTWFTPNE---EQGLTTKAQLMARIAGAMGGRAAEEFGDDEVTTG

AGGDLQQVTEMARQMVTRFGMS-NLGPISLE-SEYSEEVATRIDAQVRQLAEQGHQMARKIVQEQREVVDRL

VDLLIEKETIDGEEFRQIVAEY-

>syne4_BAA17205

-IDLDINRTPDN--SALYGLLTNLLVVAILIGLVVMVVRRSANA---RFQM-EAKTGVGFDDVAG-IDEAKE

ELQEVVTFLKQPEKFTAIGAKIPRGVLLIGPPGTGKTLLAKAIAGEAGVPFFSISGSEFVEMFVGVGASRVR

DLFKKAKENAPCLVFIDEIDAVGRQR-GVG-YG-GGNDEREQTLNQLLTEMDGFEGN---------SGIIVI

AATNRPD-VLDLALLRPGRFDRQVTVDYPDVQGRELILAIHAQNK--KLH-----VQLAAIARRTPGFTGAD

LANVLNEAAIFTARRRKE-AITMAEVNDAIDRVVAGM-------EGT---PLVDSKSKRLIAYHEVGHALIG

----TLCPGHDPVEKVTLIPRGQ-AQGLTWFTPDE---DQSLMTRNQMIARIAGLLGGRVAEEFGDDEVTTG

AGNDIEKITYLARQMVTKLGMS-SLGLVALE-SEYSEDIAARIDREIQAIVTAAHQRATRIIEENRNLMDLL

VDALIDQETIEGEHFRQLVESY-

>hnep_gi_114797326

-NNVPFSVKPDTGRNNVASVLLSLLPILLIVGFVFFMMRQMQGGGT-RLLT-EKHGRVTFDDVAG-VDEAKE

ELQEIVEFLQDPSKFQRLGGKIPKGALLVGPPGTGKTLLARAVAGEAGVPFFTISGSDFVEMFVGVGASRVR

DMFEQAKRSAPCIIFIDEIDAVGRSR-GAG-LG-GGNDEREQTLNQLLVEMDGFEAN---------EGIIIM

AATNRPD-VLDPALLRPGRFDRQVTVGNPDIIGREKILRVHMRNV--PLA-----VETKTIARGTPGFSGAD

LANLVNEAALLAARRGKR-VVAMQEFEDAKDKVLMGP-------ERR--SMVMSEKEKILTAWHEAGHAVVA

----MKVPAADPVHKATIIPRG-RALGMVMQLPED---DKLSMSKVEMTSRLAIIMGGRVAEEFGDDNVTAG

AASDIQQATRLARAMVTRWGYSDIIGPVDYG-SHISEDTSRKIEEEVRKLIEKGKEDARQVMTEFRKEWEAI

ATGLLEFETLTGEEIAGLLKGT-

>tequ_gi_31 9778415

-NGVRVTARPPEKPSFLLSALISWFPMILLIGVWIFFMRQMQGGGK-RMLD-EKNNNITFADVAG-VDEAKE

DVQEIVEFLKDPSKYQRLGGRIPRGVLMVGPPGTGKTLLAKAIAGEAKVPFFTISGSDFVEMFVGVGASRVR

DMFENAKKHSPCIIFIDEIDAVGRQR-GAG-LG-GGNDEREQTLNQMLVEMDGFESG---------QSVIVI

AATNRPD-VLDPALLRPGRFDRQVVVNLPDVRGRTQILKVHMRKV--PLA-----VDPSILARGTPGFSGAD

LANLVNEAALFAARRNGR-TVDMIDFEKAKDKIIMGA-------ERK--SMVMPEEERKNTAYHESGHAVVA

----YVLPKTDPVHKVTIIPRG-RALGVTMQLPEE---DRYSMDKERLLNMIAVLFGGRIAEEMN--QMTTG

ASNDFERATSIARDIVTRYGMTDSLGPMVYA-THVSEATMQQVDKEIRRIIDEQYKVARDIIESNREKIEVM

AKALLEWESIDSDQIKEIMDGK-

>nitr_gi_325982671

-AGVIVEAKPEEEPSMLMSIFISWFPMLLLIAVWIFFMRQMQGGGRNRMLD-KSANTVTFNDVAG-CEEAKE

EVAELVEFLRDPTKFQKLGGRIPRGVLMVGSPGTGKTLLARAIAGEAQVPFFSISGSDFVEMFVGVGASRVR

DMFEQAKKHAPCIIFIDEIDAVGRQR-GAG-LG-GGNDEREQTLNQLLVEMDGFEGA---------MGVIVI

AATNRPD-VLDPALLRPGRFDRQVTVPLPDIRGREQILHVHMRKV--PLS-----VKADILARGTPGMSGAD

LANLVNEAALFAARSNKR-LVDMDDFERAKDKIFMGA-------ERR--SMVMPEHERRNTAYHESGHAVVA

----QLLPKTDPVHKVTIIPRG-RALGVTMQLPTE---DRFSMEREEILQRISVMFGGRIAEEMK--QMTTG

ASNDFERATDLARQMVTQWGMSDELGPMVYG-KNMSEATMQKVDAEVRRIVDEQYAIARKLIEENKDKIEAM

TQALLEWETIDSDQIKDIMEGR-

>ecol_AP_003721

-KNVKVVGEPPEEPSLLASIFISWFPMLLLIGVWIFFMRQMQGGGG-RMLT-EDQIKTTFADVAG-CDEAKE

EVAELVEYLREPSRFQKLGGKIPKGVLMVGPPGTGKTLLAKAIAGEAKVPFFTISGSDFVEMFVGVGASRVR

DMFEQAKKAAPCIIFIDEIDAVGRQR-GAG-LG-GGHDEREQTLNQMLVEMDGFEGN---------EGIIVI

AATNRPD-VLDPALLRPGRFDRQVVVGLPDVRGREQILKVHMRRV--PLA-----IDAAIIARGTPGFSGAD

LANLVNEAALFAARGNKR-VVSMVEFEKAKDKIMMGA-------ERR--SMVMTEAQKESTAYHEAGHAIIG

----RLVPEHDPVHKVTIIPRG-RALGVTFFLPEG---DAISASRQKLESQISTLYGGRLAEEYGPEHVSTG

ASNDIKVATNLARNMVTQWGFSEKLGPLLYA-KHMSDETARIIDQEVKALIERNYNRARQLLTDNMDILHAM

KDALMKYETIDAPQIDDLMARR-

>vcho_NP_230286

-QDVKVQGTPPEEQSLLGTIFISWFPMILLIGVWIFFMRQMQGGGG-RMMS-EDQIKTTFSDVAG-CDEAKE

DVKELVDYLRDPSRFQKLGGKIPTGVLMVGPPGTGKTLLAKAIAGEAKVPFFTISGSDFVEMFVGVGASRVR

DMFEQAKKASPCIIFIDEIDAVGRQR-GAG-VG-GGHDEREQTLNQMLVEMDGFEGN---------EGIIVI

AATNRPD-VLDPALLRPGRFDRQVVVGLPDVRGREQILKVHMRKV--PLA-----VEPSLIARGTPGFSGAD

LANLVNEAALFAARGNKR-NVSMVEFELAKDKIMMGA-------ERR--SMVMSEEIKESTAYHEAGHAVVG

----RLVPEHDPVYKVSIIPRG-RALGVTMYLPEQ---DRVSMSKQHLESMISSLYGGRLAEEYGKEKVSTG

ASNDIERATEIARKMVTQWGFSEKLGPMLYA-KHMSDDTAKLIDDEVRQIIDRNYERARQIIMDNMDIMHAM

KDALMKYETIDAGQIDDLMARK-

>ther_gi_337288901

-KNIKIVAKPENQNSWLTSFLISWLPFLILIVLWIVFLKQLQ-PSN-KLIK-EGNTKVTFNDVAG-IEEVKE

ELQDIVEFLKNPQKFTKLGARIPKGILLVGPPGTGKTLLAKAIAGEAGVPFFSISGSDFVEMFVGVGAARVR

DLFSQAKAHAPCIIFIDEIDAVGRQR-GAG-LG-GGHDEREQTLNQLLVEMDGFDTG---------EGIVVL

AATNRPD-ILDPALLRPGRFDRQVYVPPPDVNGREAILRLYAKKF--KVD-----IDFKAIAKGTPGFTGAD

LENMLNEAALIAAKKGKE-KIEIEDLEEAKDKILIGK-------ERK--GIVLNEEERKIIAYHEAGHALVA

----YYLPDPDPVHKISIIPRG-QALGVTQQLPLD---DRHIYTEDYLLKKITVLLGGRVSEEFN--KVSSG

AQDDLKRATQIARKMVCNWGMSKKLGPVTFG-KDFSEETARIIDEEVKNIILSCYEKAKTILNQYLHKLHKI

AQTLLEEETIDADRFKLILEGH-

>tmar_NP_228390

-KGIKVSGERSGSSSFWINVLGTLIPTILFIVVWLFIMRSLSGRNN-TMYKPSGNKRVTFKDVGG-AEEAIE

ELKEVVEFLKDPSKFNRIGARMPKGILLVGPPGTGKTLLARAVAGEANVPFFHISGSDFVELFVGVGAARVR

DLFAQAKAHAPCIVFIDEIDAVGRHR-GAG-LG-GGHDEREQTLNQLLVEMDGFDSK---------EGIIVM

AATNRPD-ILDPALLRPGRFDKKIVVDPPDMLGRKKILEIHTRNK--PLA-----VNLEIIAKRTPGFVGAD

LENLVNEAALLAAREGRD-KITMKDFEEAIDRVIAGP-------ARK--SKLISPKEKRIIAYHEAGHAVVS

----TVVPNGEPVHRISIIPRGYKALGYTLHLPEE---DKYLVSRNELLDKLTALLGGRAAEEFG--DVTSG

AANDIERATEIARNMVCQLGMSEELGPLAWG-RNYSEEVASKIDEEVKKIVTNCYERAKEIIRKYRKQLDNI

VEILLEKETIEGDELRRILSEE-

>athal8_AAD30220

-AGVEVDLLQKRQIHYFMKVFIALLPGILILWFIRESAMLLLITSKRLPVGDVSETKSMYKEVVLG-GDVWD

LLDELMIYMGNPMQYYEKDVAFVRGVLLSGPPGTGKTLFARTLAKESGLPFVFASGAEFTD-SEKSGAAKIN

EMFSIARRNAPAFVFVDEIDAIAGRHARKD-------PRRRATFEALIAQLDGEKEKTGIDRFSLRQAVIFI

CATNRPD-ELDLEFVRSGRIDRRLYIGLPDAKQRVQIFGVHSAGKN---------------LAEDIDFGKAN

IRNLVNEAAIMSVRKGRSYIYQQDIVDVLDKQLLEGMGVLLTEEEQQKCEQSVSYEKKRLLAVHEAGHIVLA

----HLFPRFDWHAFSQLLPGGK-ETAVSVFYPREDMVDQGYTTFGYMKMQMVVAHGGRCAER---DNVTDG

GKDDLEKITKIAREMVISPQLVKKIGMVDLPPAEMSVEVSELFTRELTRYIEETEELAMNALRANRHILDLI

TRELLEKSRITGLEVEEKMKDL-
